# An Unusual Component of the Multistep Phosphorelay from Tea Plant (*Camellia sinensis* L.)

**DOI:** 10.3390/ijms27104253

**Published:** 2026-05-10

**Authors:** Ekaterina M. Savelieva, Dmitry V. Arkhipov, Georgy A. Romanov, Olga G. Leonova, Vladimir I. Popenko, Natalia V. Zagoskina, Sergey N. Lomin

**Affiliations:** 1 Timiryazev Institute of Plant Physiology, Russian Academy of Sciences, Botanicheskaya Str. 35, 127276 Moscow, Russia; savelievaek@yandex.ru (E.M.S.); hotdogue@yandex.ru (D.V.A.); g_a_rom@mail.ru (G.A.R.); nzagoskina@mail.ru (N.V.Z.); 2 Engelhardt Institute of Molecular Biology, Russian Academy of Sciences, Vavilov Str. 32, 119991 Moscow, Russia; leonova-kozma@mail.ru (O.G.L.); popenko@eimb.ru (V.I.P.)

**Keywords:** cytokinin, phosphotransmitter, MSP, transmembrane domain, cytokinin receptor, endoplasmic reticulum, *Camellia sinensis*

## Abstract

Recently, the existence of a new class of plant phosphotransfer proteins (HPts) with transmembrane (TM) domains was predicted by a large-scale bioinformatics method. These non-canonical proteins belong to the multistep phosphorelay (MSP) signal transduction system. The gene for one of these predicted TM-HPt was first cloned from tea (*Camellia sinensis* L.) plant cells. The membrane localization of the encoded protein (TM-CsHPt1) was confirmed using confocal microscopy and immunoblotting. These proteins were detected in the endoplasmic reticulum-enriched but not plasma membrane-enriched fractions. Using the BiFC method, the ability of TM-CsHPt1 to homodimerize was shown, similar to classical soluble HPt. However, heterodimerization between canonical and non-canonical CsHPts was not detected. Furthermore, TM-CsHPt1 was capable of specific interaction with the Arabidopsis cytokinin (CK) receptor AHK3, but not its paralogs AHK2 and AHK4. The obtained data are compatible with the involvement of TM-CsHPt1 in CK signaling (which utilizes the MSP system), possibly through a suggested non-canonical membrane branch. In addition, the key components of the CK signaling system in *C. sinensis* were uncovered and characterized by bioinformatics and phylogenetic analysis. The putative functions of the predicted MSP membrane branch in the tea plant are discussed.

## 1. Introduction

The multistep phosphorelay (MSP) is a conserved signaling mechanism that allows plants to grow and develop in an unstable environment [1]. MSP originated from the two-component system (TCS), a common mechanism for signal transduction in prokaryotes. In the classical TCS, signal perception causes autophosphorylation of the histidine kinase (HK) protein at its conserved histidine (His) residue. Then the response regulator protein (RR) accepts “hot” phosphate onto its conserved aspartate (Asp) residue directly from the HK [2]. When signaling pathways (both in prokaryotic and eukaryotic organisms) contain more than two elements, they are termed MSP. MSP in plants includes three key protein types. In addition to HKs and RRs, phosphotransfer proteins, or histidine-containing phosphotransmitters (HPts), participate in signal transduction. Since HKs involved in MSP contain an additional receiver domain (RD) with the conserved Asp residue, the His-containing HPts participate in the phosphoryl transfer through His-Asp-His-Asp residues [1].

In the prokaryotic (*E. coli*) variant of the MSP system, the HPt (termed RcsD) is a multidomain and transmembrane (TM) protein [3,4]. However, in plants, classical HPts are single-domain small soluble proteins [5,6,7,8]. In the commonly accepted MSP model, HPts play a crucial role, constantly shuttling between the cytosol and the nucleus [6] and bridging membrane-bound HKs in the plasma membrane (PM) or endoplasmic reticulum (ER) with RRs in the nucleus [6,9,10]. In plants, these nuclear acceptors are mainly B-type RRs (RRBs), which are transcription factors that affect target gene transcription and play a positive role in signaling [10,11], or A-type RRs (RRAs), which lack a DNA-binding motif and negatively regulate signaling [12]. In addition, pseudo RRs (PRRs) are distinguished separately, which are largely similar to RRAs but lack the conserved phosphoaccepting Asp [13].

Plant HPts are mostly the downstream intermediate participants of the signal transduction [14], which are represented by small families [9,15]. Considering that plant MSP mediates several signal transduction pathways, including cytokinin (CK) signaling [16,17,18], partially ethylene signaling [19,20,21], and osmosensing [22,23,24], HPts exhibit high promiscuity in their interactions between both HKs and RRs, acting as a signaling hub [25,26,27,28,29].

The structure of canonical HPts is highly conserved [30]. The most conserved regions are located near the phosphoaccepting His residue [31,32] and are responsible for interaction with HKs [17]. However, there is a subset of proteins, pseudophosphotransmitters (PHPs), which are members of the HPt family but lack the phosphoaccepting His [5,33]. They interact with both HKs and RRs but cannot phosphorylate or be phosphorylated [8,26,33]. Most HPts act as positive regulators of the MSP [34], while PHPs and AHP4 orthologs are negative regulators [8,35].

Contrary to the generally accepted scheme of plant MSP and the known functions of HPts, our recent large-scale bioinformatics study predicted the existence of a new class of plant phosphotransfer proteins—transmembrane HPts (TM-HPts). The TM structure suggests unknown functions of these proteins that are not typical for canonical plant HPts [32]. Previously, we identified almost a hundred such potential TM-HPts belonging to more than 60 plant species. Of these, about 40 proteins contain both a conserved His and an intact phosphorylation motif in the HPt domain. Another 13 proteins can be considered PHPs because they have the HPt domain but lack the conserved His. According to available transcriptomic data, most transcripts encoding potential functional TM-HPt are likely produced in vivo [32].

Although we found two dozen genes in 13 plant species that express mRNA version(s) encoding only TM-HPts, most putative TM-HPts are encoded as one of the splice forms of a particular gene along with classical soluble variants [32]. This complicates cloning the desired proteins from the plant material, as the expression of a required splice form can be organ- and/or stage-specific. Furthermore, if the short RNA sequence with a start AUG codon of a non-TM protein is included in a longer TM protein coding sequence, then, in the case of an alternative translation initiation, both long (TM-containing) and short (without TM) protein versions can be formed on a single template.

After a detailed analysis, we have chosen the XP_028076088.1 protein (according to NCBI) from *Camellia sinensis* for experimental searching of TM-HPts in plant cells. Previously, we have demonstrated the functionality of the TM domain experimentally from this protein [36]. The present work aims to study the expression, properties, localization, and interactions of tea TM-HPts in planta, providing insight into possible functions of such proteins in CK signaling.

## 2. Results

### 2.1. Expression of Putative Transmembrane Phosphotransmitter Gene in C. sinensis Cells

A predicted TM-containing phosphotransfer protein XP_028076088.1 (TM-CsHPt1) from *C. sinensis* seems to be suitable for experimental study of TM-HPts. It possesses a 15 amino acid (aa) phosphorylation motif including a phosphorylatable His residue, which is characteristic of active canonical HPts [32]. The molecular weight of TM-CsHPt1, as well as the position, size, and number (single) of its TM domain, are typical for putative TM-containing phosphotransfer proteins [32].

**Figure 1:**
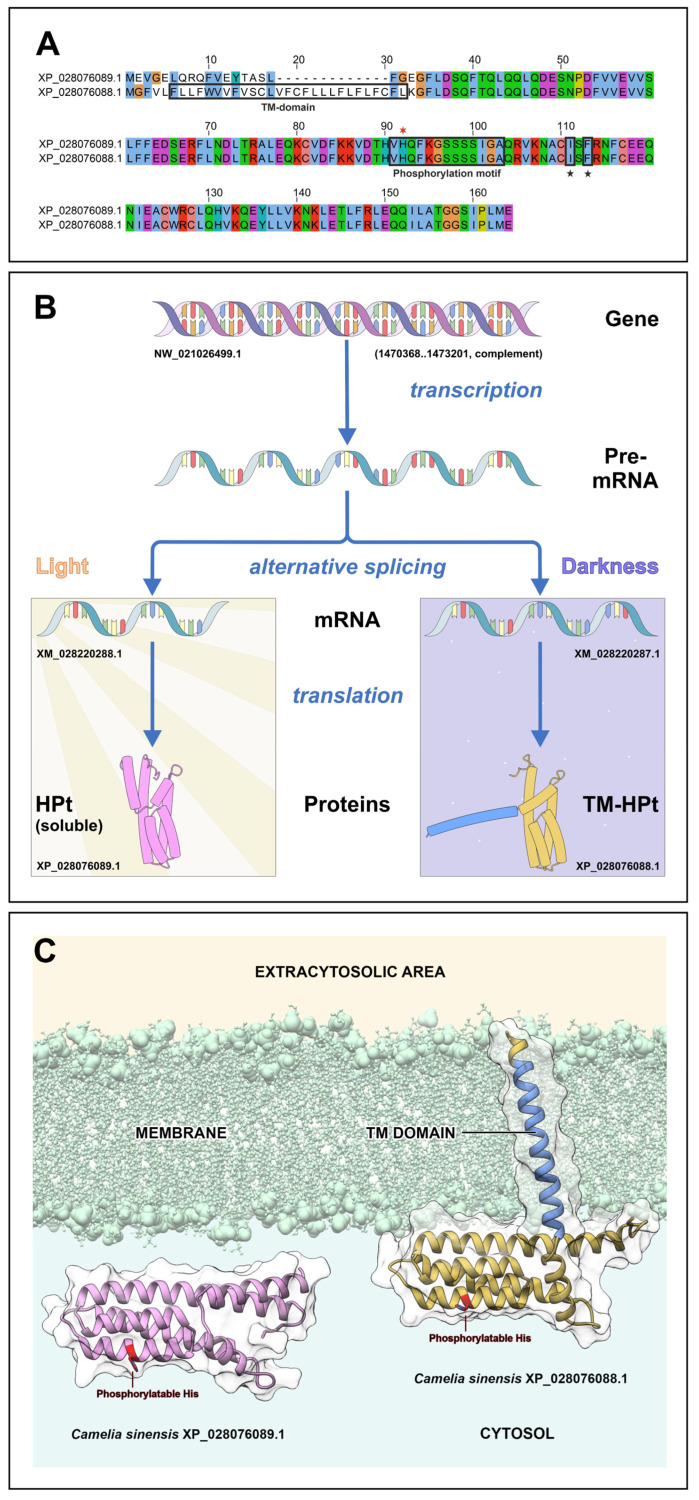
Main features and biosynthesis of CsHPt1 isoforms in *C. sinensis* cells. (**A**) Sequence alignment of the CsHPt1 isoforms. Amino acids (aa) in black frames belong to the domain or motif as indicated under them. Red asterisk designates the phosphoaccepting His; black asterisk designates distant aa of the phosphorylation motif. (**B**) General scheme for CsHPt1 biosynthesis by alternative splicing. TM-CsHPt1 appears when grown in the dark. (**C**) Molecular models of TM-CsHPt1 and sol-CsHPt1. The TM segment is highlighted in blue, the phosphoaccepting histidine in red.

The cognate *C. sinensis* gene LOC114278265 was predicted to encode two phosphotransfer proteins due to alternative pre-mRNA splicing: the longer version (164 aa) corresponds to TM-CsHPt1, while the shorter version (151 aa) represents a canonical soluble HPt (termed here sol-CsHPt1) without a TM domain (Figure 1A). Importantly, these two mRNA splice forms differ in their first exons, allowing us to discriminate between them using PCR with specific primers (see Section 4 and Table S1).

To analyze the pattern of *TM-CsHPt1* expression, we searched for the corresponding transcript XM_028220287.1 in plant samples from *C. sinensis*. The cognate transcript XM_028220288.1 encoding sol-CsHPt1 was used as a control for gene activity. Total RNA was isolated from expanded leaves and leaf buds of *C. sinensis* plants grown in a greenhouse, as well as from callus tissues cultivated in constant darkness or under a 16/8 h light/dark cycle. cDNAs were synthesized from total RNAs, and coding sequences of the two close HPt forms were PCR-amplified and cloned. Notably, the *sol-CsHPt1* DNA sequence was cloned from all the samples except the dark-grown callus, while *TM-CsHPt1* DNA was found only in this dark-grown source (Figure 1B).

### 2.2. Subcellular Localization of Putative Transmembrane and Canonical Phosphotransfer Proteins CsHPt1 from C. sinensis

Molecular modeling predicted strong membrane binding of the TM-CsHPt1 protein [32] (Figure 1C). To verify this prediction, coding DNAs of the two CsHPt1 isoforms (TM-CsHPt and sol-CsHPt) were put under the control of the constitutive 35S CaMV promoter and fused at the 3′-end to the eGFP reporter sequence. In addition to these constructs, nuclear and PM markers were created. The P88L mutated auxin signaling inhibitor IAA17 was used as a nuclear marker [37]. As a PM marker, the histidine kinase AHK1 sensor module from Arabidopsis, flanked on both sides by TM segments, was used [38]. Both marker sequences were fused to the mCherry fluorescent reporter. In addition, a construct *TM-AHK1-TM–eGFP* was generated, allowing the use of immunoblotting with anti-GFP antibodies.

**Figure 2:**
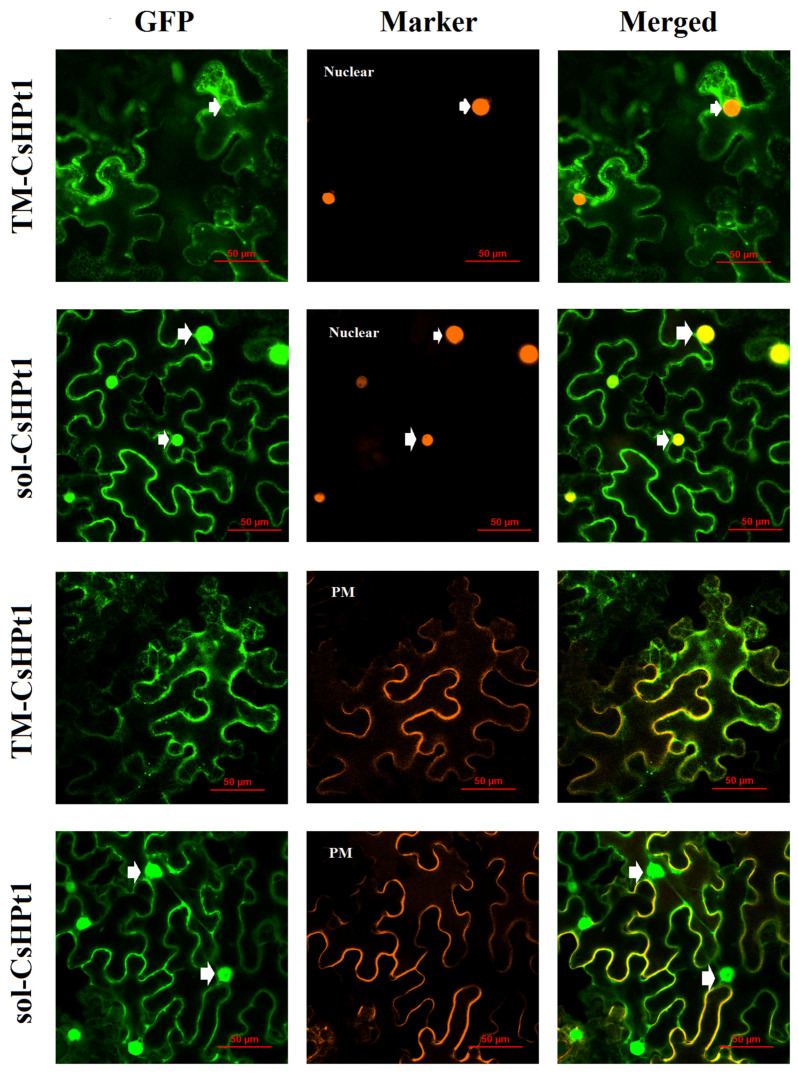
Co-localization of TM and soluble HPt isoforms of *C. sinensis* with the PM and nuclear marker proteins, visualized by confocal microscopy. Bars = 50 μm. White arrows indicate nuclei. Inscriptions indicate proteins encoding by: TM-CsHPt, *TM-CsHPt–eGFP* construct; sol-CsHPt1, *sol-CsHP1t–eGFP* construct. GFP, the column with eGFP fluorescence channel (shown in green); Marker, the column with mCherry fluorescence channel (shown in red); Merged, the column with merged channel. Yellow color indicates co-localization of the CsHPt proteins and the markers.

Each of the five resulting constructs (*p35S–TM-CsHPt1–eGFP*, *p35S–sol-CsHPt1–eGFP*, *p35S–IAA17_P88L–mCherry*, *p35S–TM-AHK1–mCherry*, and *p35S–TM-AHK1–eGFP*) was cloned into an expression vector pB7FWG2 [39], transferred into *Agrobacterium tumefaciens* (*Rhizobium radiobacter*) strain GV3101, and transiently expressed in *Nicotiana benthamiana* leaves [40]. The two isoforms of CsHPt1 were expressed either separately or co-expressed in combination with each of the markers. Five days after inoculation, the localization of CsHPt1 proteins was analyzed by confocal microscopy (Figure 2).

Results showed that sol-CsHPt1–eGFP co-localized with the nuclear marker and partially with the PM marker. This variant also showed individual strands crossing the cell segments observed only in the eGFP fluorescence channel (Figure 2 and Figure S1).

Unlike canonical sol-CsHPt1, the fluorescent signal of the TM-CsHPt1–eGFP surrounded the nucleus without co-localization with the nuclear marker. As in the case of sol-CsHPt1, there was partial co-localization of TM-CsHPt1 with a PM marker. In contrast to the single strands observed in the variant with sol-CsHPt1, the fluorescent signal of TM-CsHPt1 formed a dense net inside the cell. This net did not co-localize with the markers used (Figure 2 and Figure S1). The subcellular pattern of the fluorescent proteins did not change depending on whether the generated constructs were expressed separately or together.

**Figure 3:**
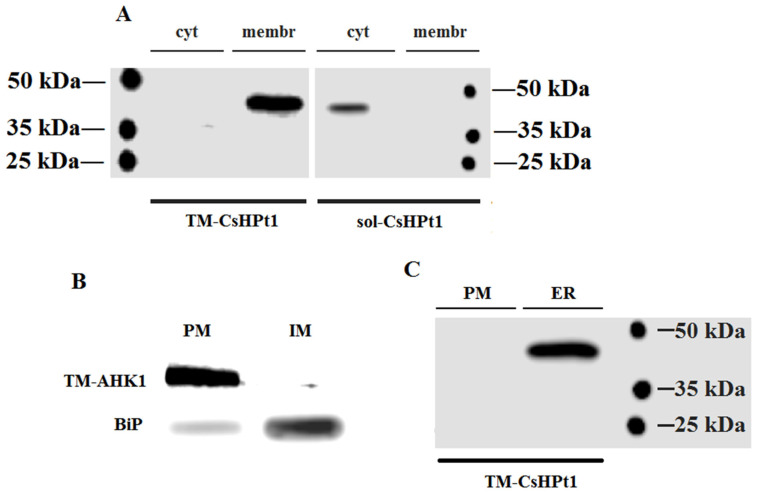
(**A**) Immunological detection of soluble and transmembrane CsHPt1 in cytosol and microsomal membrane fractions obtained from tobacco leaves overexpressing *TM-CsHPt1–eGFP* and *sol-CsHPt1–eGFP* DNA constructs. TM-CsHPt1, transmembrane isoform of CsHPt1; sol-CsHPt1, soluble isoform of CsHPt1; cyt, cytosol fraction; membr, membrane fraction. (**B**) The purity of membrane fractions obtained by aqueous two-phase partitioning from tobacco leaves overexpressing *TM-AHK1–eGFP* tested by immunoblotting using antibodies against GFP and BiP. PM: plasma membrane-enriched fraction; IM: inner membrane-enriched fraction. (**C**) Western blotting of transmembrane CsHPt1 in membranes obtained from tobacco leaves overexpressing TM-CsHPt1–eGFP and fractionated by aqueous two-phase partitioning. PM: plasma membrane-enriched fraction; ER: endoplasmic reticulum-enriched fraction. Positions of protein bands on the gels (**A**,**C**) are in accordance with the calculated protein fusion biomasses, 47.6 kDa and 45.9 kDa for TM-CsHPt1–eGFP and sol-CsHPt1–eGFP, respectively.

Apart from microscopy, the localization of both CsHPt1 isoforms was verified by immunoblotting. Homogenates were obtained from tobacco leaves transiently transformed with *p35S–TM-CsHPt1–eGFP* and *p35S–sol-CsHPt1–eGFP*. The homogenates were separated by differential centrifugation into cytosolic and total microsomal membrane fractions. The presence of CsHPts in both fractions was checked by immunoblotting using anti-GFP antibodies (Figure 3A).

As was expected, the canonical sol-CsHPt1 protein was present only in the cytosolic fraction. In contrast, TM-CsHPt1 was detected in the membrane fraction and was totally missing in the cytosol.

The total membrane pool isolated from leaf cells co-transformed with TM-CsHPt1–eGFP and TM-AHK1-TM–eGFP was separated into fractions enriched with the PM or ER microsomes by the aqueous two-phase polymer partitioning, mainly as described [41]. The enrichment of the obtained fractions with PM or ER microsomes was proven by immunoblotting with antibodies against eGFP or BiP chaperone protein, respectively. TM-AHK1-TM–eGFP was almost entirely found in the microsomes, presumably enriched in the PM, whereas in the fractions enriched in intracellular membranes, the content of the BiP marker greatly prevailed (Figure 3B).

The occurrence of TM-CsHPt1–eGFP in both membrane fractions was similarly checked by immunoblotting (Figure 3C). According to the blotting results, TM-CsHPt1 was detected only in the fraction of inner membranes, which are greatly enriched in the ER.

### 2.3. Protein–Protein Interactions of the Transmembrane CsHPt1

The role of TM-HPts in the MSP system should primarily depend on their interactions with other MSP participants. Although yet unexplored signaling pathways may be discovered, the highly conserved phosphorylation motif in many identified putative TM-HPts [32] may suggest their interaction with already known MSP components. Canonical soluble HPts exhibit high promiscuity, interacting not only with all key components of MSP (HKs, RRs, and HPts) but also with other proteins, for example, Cytokinin Response Factors and some molecular switches [14]. In this study, we investigated protein–protein interactions of TM-CsHPt1 with HKs, canonical sol-CsHPt1, and with each other (homodimerization). Among HKs involved in MSP, CK receptors were selected, which typically interact with all HPts in different plant species [14].

To investigate putative interactions between CsHPt1 isoforms and CK receptors in planta, we performed bimolecular fluorescence complementation (BiFC) analysis upon transient expression of these proteins in *N. benthamiana* leaves. Given the highly conserved structure of CK receptors [42,43] and their ability to trigger phosphorelay with phylogenetically very distant HPts [44], the full set of Arabidopsis receptors (AHK2–4) [45,46,47] was used for this work.

**Figure 4:**
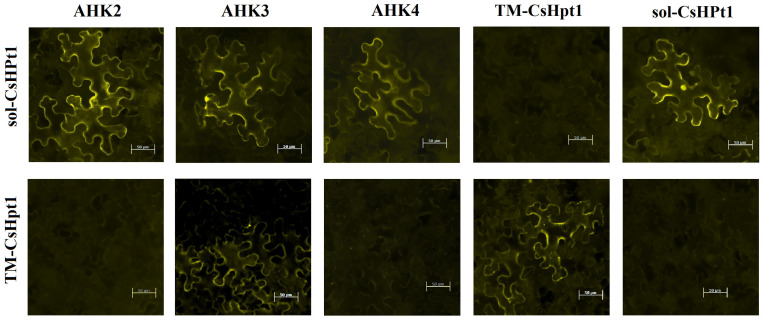
Interactions of CsHPt1 isoforms with Arabidopsis CK receptors in planta determined by BiFC and visualized by fluorescence microscopy (vectors *pSPYCE-35S–AHK2*–*4*, *pSPYNE-35S–TM-CsHPt1*, *pSPYCE-35S–sol-CsHPt1,* and *pSPYNE-35S–sol-CsHPt1*). eYFP fluorescence is shown in yellow. Bars = 50 μm.

The selected genes were inserted into *pSPYNE-35S* and *pSPYCE-35S* vectors, then pairwise co-expressed in tobacco leaves, and protein–protein interactions were tested using fluorescence microscopy. The corresponding negative controls were also performed (Figure 4 and Figure S2).

As expected, no fluorescence was registered in variants with negative controls, while specific eYFP fluorescence was detected for all AHKs + sol-CsHPt1 combinations. Accordingly, the eYFP signal was detected in the sol-CsHPt1 homodimerization variant. In combinations including TM-containing phosphotransfer protein (TM-CsHPt1), fluorescence was observed only in the TM-CsHPt1 + TM-CsHPt1 (homodimerization) and AHK3 + TM-CsHPt1 variants. No interaction was observed between TM-CsHPt1 and sol-CsHPt1. The resulting fluorescence output for each protein combination was the same regardless of the vector (*pSPYNE-35S* or *pSPYCE-35S*) used to express individual proteins (Figure 4 and Figure S2).

### 2.4. The Influence of TM-CsHPt1 on CK Signaling in Planta

Several hypotheses have been put forward regarding the possible influence of functional TM-HPts on the MSP and, in particular, CK signal transduction—both positive and negative [32]. To test the potential negative impact of TM-HPt on CK signaling, the *pARR5–mCherry* and *pARR5–eGFP* constructs were generated, in which the reporter gene was fused to the promoter of the CK primary response gene. If the CK signaling in the cell is suppressed, the expression of these constructs is expected to be downregulated. Constructs *pAHP3–AHP6–mCherry* and *p35S–eGFP* were also created as controls. The *AHP6* gene, encoding a negative regulator of CK signaling, is hardly expressed under strong promoters in plant cells. Therefore, we placed it under the control of a moderate promoter of the related *AHP3* gene.

**Figure 5:**
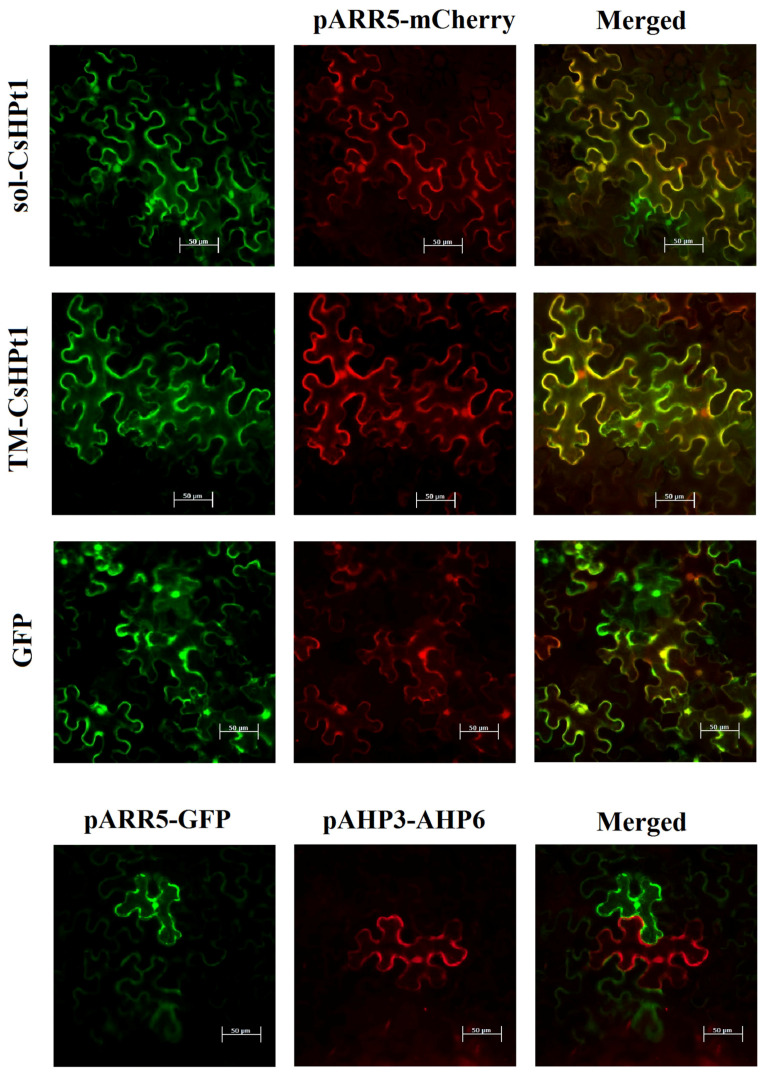
Co-expression of TM and soluble isoforms of *C. sinensis HPt* genes with the *pARR5-mCherry* reporter construct, visualized by fluorescence microscopy. Inscriptions indicate: sol-CsHPt1, *sol-HPt–eGFP* construct; TM-CsHPt1, *TM-HPt–eGFP* construct; GFP, *p35S–eGFP* construct; pARR5–mCherry, *pARR5–mCherry* construct; pARR5–GFP, *pARR5–eGFP* construct; pAHP3–AHP6, *pAHP3–AHP6–mCherry* construct. The left column: eGFP fluorescence channel (shown in green); the middle column: mCherry fluorescence channel (shown in red); the right column (Merged): merged channel. Yellow color indicates co-expression of the *GFP* and *mCherry*. Bars = 50 μm.

At first, the *p35S–TM-CsHPt1–eGFP*, *p35S–sol-CsHPt1–eGFP*, and control *pAHP3–AHP6–mCherry* constructs were transiently expressed in tobacco leaves for three days. Then leaves with the confirmed expression were further inoculated with an Agrobacterium suspension carrying *pARR5–mCherry* or (in the case of leaves expressing *pAHP3–AHP6–mCherry*) *pARR5–eGFP*, and the constructs were co-expressed for an additional three days. The resulting expression was assessed using fluorescence microscopy (Figure 5).

The preceding expression of *p35S–TM-HPt–eGFP* or *p35S–sol-HPt–eGFP* had no visible effect on the expression of the *pARR5–mCherry*. A similar pattern was observed in the negative control variant *p35S–eGFP* + *pARR5–mCherry*. However, in the control co-transformation with *pAHP3–AHP6–mCherry* and *pARR5–eGFP*, there was no visible co-expression of eGFP and mCherry in the same cells.

### 2.5. Bioinformatics Characteristic of the Main Components of C. sinensis CK Signaling System

The data obtained in our experiments indicated a very likely involvement of *C. sinensis* TM-HPt1 in CK signal transduction. This piqued our interest in the overall organization of CK signaling in the tea plant. We first set out to determine whether there is any peculiarity of key CK signaling components that could directly interact with the membrane phosphotransfer protein. These components include receptors and response regulators. Therefore, we performed a bioinformatics analysis to characterize the key proteins of the CK signaling system in the tea plant.

#### 2.5.1. Putative CK Receptors of *C. sinensis*

Bioinformatics analysis predicts the presence of six hybrid HKs in *C. sinensis*, similar in structure to known CK receptors. Like other CK receptors of angiosperms, they are large proteins (978 to 1250 aa) with predicted 2–4 TM domains and consist of 10 to 13 protein-coding exons (Table S2).

To clarify the evolutionary relationships of putative CK receptors in *C. sinensis* with CK receptors of other angiosperm species, we performed a phylogenetic analysis using the protein sequences. To generate the evolutionary tree, we used sequences from the eudicot *Arabidopsis thaliana*, and the monocot *Oriza sativa* japonica group, as well as *Amborella trichopoda* and *Nymphaea colorata*. *A. trichopoda* was chosen as one of the most primitive representatives of the angiosperm clade. However, since this species lacks ortholog(s) of the Arabidopsis CK receptor AHK4, we also used *N. colorata*, another ancient species of the angiosperm group.

**Figure 6:**
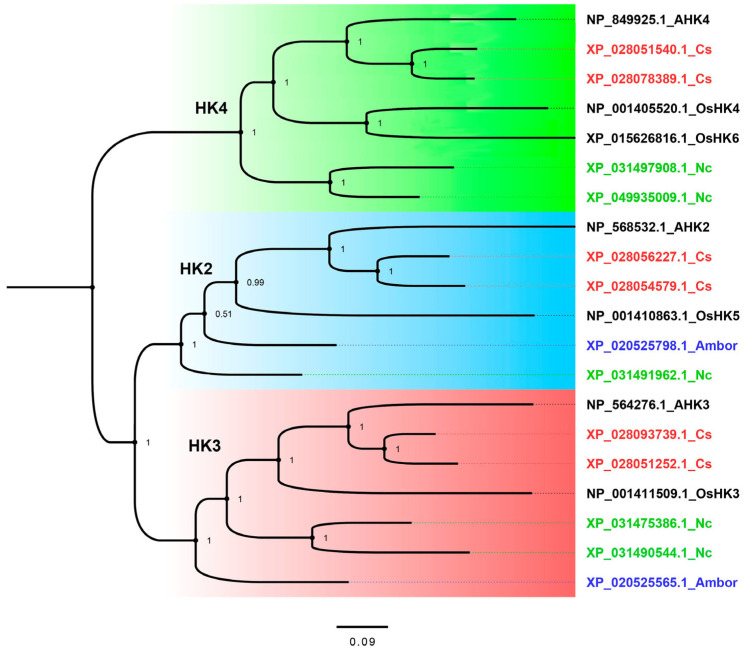
Phylogenetic tree of the CK receptors based on the protein sequences. This tree was constructed using MrBayes-3.2.7. The column on the right provides protein accession IDs and abbreviated plant species names or protein trivial names. Ambor, *Amborella trichopoda*; AHK2–4, Arabidopsis histidine kinase 2–4; Cs, *Camellia sinensis*; Nc, *Nymphaea colorata*; OsHK3–6, *Oriza sativa* histidine kinase 3–6. The numbers at the base of the branches represent Bayesian posterior probabilities.

The resulting phylogenetic tree includes three clades, which, based on their orthologs in the model plant *A. thaliana*, were named HK2, 3, and 4 (Figure 6).

*C. sinensis* has two representatives of putative CK receptors in each clade. The detailed domain structure of uncovered tea CsHK2–4 is consistent with the established structural features of canonical CK receptors [42].

The extracytosolic N-terminus part of the proteins represents a ligand-recognizing sensor module (SM), consisting of a dimerization interface (DI) and a CHASE (Cyclase/Histidine kinase Associated SEnsory) domain. The cytosolic part of the receptor includes the HK-dimerization (HisKA), H-ATPase, receiver-like (REC-like) domains, and receiver domain (RD) [27,43]. SM is flanked by TM segments; the number of upstream SM is variable, but only one TM segment lies downstream RD. For putative tea CK receptors CsHKs2, 3, and 4, the number of upstream TM segments is equal to three, two, and one, respectively (Figure S3). This corresponds to the general rule reported previously [42].

#### 2.5.2. Putative Phosphotransmitters of *C. sinensis*

**Figure 7:**
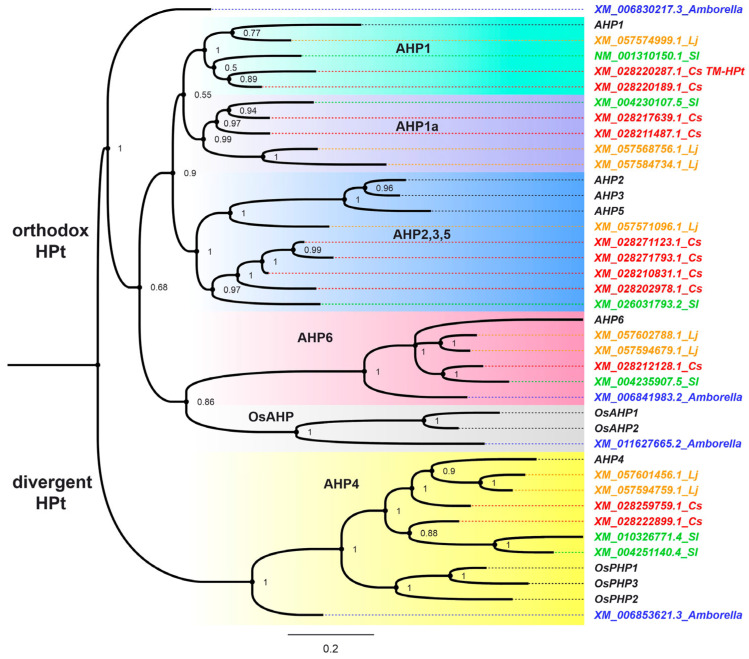
Phylogenetic tree of the plant HPts based on the RNA coding sequences. This tree was constructed using MrBayes-3.2.7. The column on the right provides transcript accession IDs and abbreviated plant species names or protein trivial names. *Amborella*, *Amborella trichopoda*; *AHP1-6*, *Arabidopsis Histidine Phosphotransmitter1-6*; *Cs*, *Camellia sinensis*; *Lj*, *Lotus japonicus*; *OsAHP1-2*, *Oriza sativa Authentic Histidine Phosphotransmitter1-2*; *OsPHP1-3*, *Oriza sativa Pseudo Histidine Phosphotransmitter1-3*; *Sl*, *Solanum lycopersicum*. The numbers at the base of the branches represent Bayesian posterior probabilities.

We found 11 genes encoding putative *C. sinensis* HPts (Table S3). Using phylogenetic analysis, we attempted to determine their position among various HPt groups. It is worth noting that the phylogenetic tree of such small and highly similar proteins may not be relevant enough. Therefore, to obtain confidential results, the trees based on RNA coding sequences were generated (Figure 7).

Using evolutionarily significant species of angiosperms allowed us to obtain a more reliable picture of the distribution of proteins of a given gene family across phylogenetic clades, as the tree is constructed for the entire, relatively large, but morphologically and physiologically distinct group of living organisms, representing a specific major stage in plant evolution. An important criterion for the quality of the tree construction is the consistency of the distribution of individual proteins within the tree without obvious contradictions with the basic concepts of the phylogeny of the group of organisms.

The HPt tree is divided into two main branches: divergent HPts with orthologs of AHP4 and monocot PHPs (which negatively affect CK signaling) and orthodox HPts. This tree topology is similar to that of the trees obtained earlier [32,48]. Non-monocot PHP proteins are located in the orthodox HPt clade (AHP6 clade). Other clades of the orthodox HPts include only positive regulators of CK signaling.

Within the AHP4 clade, the *A. trichopoda* representative branches off first, after which the group splits into groups of monocots (PHPs of rice) and dicots. The dicot branch further divides into two clades: one contains representatives of Arabidopsis and *Lotus japonicus*, and the other contains representatives of tomato. In *C. sinensis*, two *HPts* fall into the AHP4 clade; one of them (XM_028259759.1) is on the same branch as AHP4 orthologs from Arabidopsis and *L. japonicus*, while the other (XM_028222899.1) is on the tomato branch.

As in the divergent HPt group, in the orthodox HPt branch, the first to diverge is the *A. trichopoda* representative. Two more of *A. trichopoda HPts* were found at the bases of the OsAHP and AHP6 clades. Overall, the orthodox HPt branch can be divided into five clades, designated AHP1, AHP1a, AHP2,3,5, AHP6, and OsAHP. In the obtained tree, the first three clades lack representatives of monocots. In contrast, the OsAHP clade lacks eudicots.

The orthodox HPt group contains nine CsHPt coding sequences: one in the AHP6 clade and eight in the AHP1–3,5 group of clades. Four of these eight members are in the AHP2,3,5 clade, and two pairs each are in the AHP1 and AHP1a clades. *TM-CsHPt1* falls into the AHP1 clade, where it has a non-TM paralog.

A comparison of the aligned HPts protein sequences with their exon organization reveals a general trend toward subdividing the corresponding coding sequences into six exons (Figures S4 and S5, Table S3). This is characteristic of both orthodox and divergent HPts. The exception is the AHP6 clade, which appears to have undergone a fusion of the third and fourth exons. In Arabidopsis, the *AHP6* gene has undergone a more profound transformation of its exonic structure, reducing the number of exons to three or four in different splicing variants.

**Figure 8:**
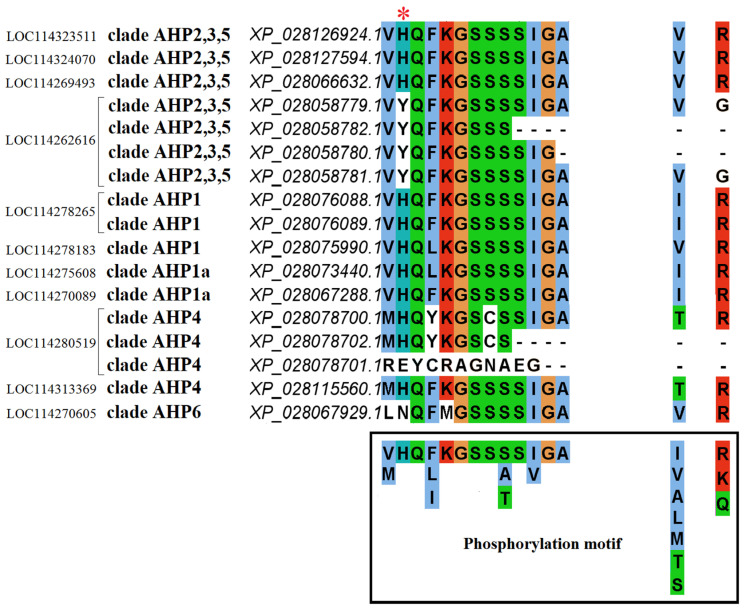
Phosphorylation motif region in the alignment of protein sequences of *C. sinensis* phosphotransmitters. Red asterisk indicates the phosphorylatable histidine. The amino acids in the phosphorylation motif of functional phosphotransmitters are shown in the black frame. The left columns provide (from left to right): gene ID, clade name (see Figure 7), protein ID.

An alignment of the protein sequences of all found putative CsHPts (Figure 8, Figures S4 and S5) revealed that only 9 of the 17 proteins possess a conserved His and the phosphorylation motif previously identified for active HPts [32]: 3 proteins in the AHP1 clade (including TM-CsHPt1 and sol-CsHPt1), 2 proteins in the AHP1a clade, 3 proteins in the AHP2,3,5 clade, and one protein in the AHP4 clade. The AHP6 clade contains CsPHP with a compromised consensus motif lacking phosphorylatable His and having two additional substitutions in this core aa sequence. The remaining 7 putative CsHPts (three in the AHP4 clade and four in the AHP2,3,5 clade) carry a significant number of substitutions in the phosphorylation motif and/or lack the active site of the phosphotransfer domain. Thus, 9 of the 17 proteins identified bioinformatically can be classified as potentially functional tea HPts; one more protein is PHP; the remaining 7 are likely not functional HPts or PHPs. It should also be noted that despite the preserved phosphorylation motif and His, two proteins from the clade AHP2,3,5 (XP_028126924.1 and XP_028127594.1) are missing a significant portion of the aa residue at the N-terminus.

#### 2.5.3. Putative B-Type Response Regulators of *C. sinensis*

In the canonical MSP scheme, HPts transmit signals to the RRs of different types, implementing both negative and positive regulation of the signaling. In the case of signal transduction to the RRBs, the signal regulation is always positive [49]. In *C. sinensis*, 15 genes that may encode proteins of this type were identified.

We performed a phylogenetic analysis of this group of proteins (Figure S6) with a special focus on tea RRBs. The resulting tree consists of six clades, according to the distribution of RBBs in the reference species *A. trichopoda* and *N. colorata*. The most divergent is the ARR13 clade. It includes ARR19, 20, and 21 of Arabidopsis. All representatives of this group are poorly studied. In tea, one protein (XP_028123619.1) belongs to this clade.

Orthodox RRB proteins are divided into five groups. In the ARR14 clade, CsRRBs are absent; the ARR1 clade includes one tea representative, while the ARR11 and 10 clades include two and three CsRRBs, respectively. A separate RR clade includes one Amborella member and 8 tea members.

Functional RRBs include a phosphate-accepting RD and a DNA-binding Myb-like domain. Alignment of aa sequences (Figure S7) revealed that one CsRRB (XP_028054587.1) in the RR-clade has a highly reduced RD. Furthermore, all 8 *C. sinensis* members of this clade exhibit significant deviations from the canonical structure of the Myb-like domain, SHAQKYF class. All members of the ARR13 group also exhibit significant deviations in this domain. In addition, the tea RRB in the ARR1 clade has a disrupted DD motif in the RD.

Thus, only five *CsRRBs* encode potentially functional proteins (Table S2). It is also worth noting that the two ARR11 orthologs in tea are neighbors along the genomic sequence and are likely the products of recent tandem duplication. Moreover, the sequence versions encoding XP_028073237.1 and XP_028073239.1 overlap.

#### 2.5.4. Putative A-Type Response Regulators of *C. sinensis*

RRA proteins lack a DNA-binding motif and consist primarily of an RD with short N- and/or C-terminal extensions. They regulate CK signaling by acting as a negative feedback loop [12].

We identified 14 genes encoding RRAs in *C. sinensis*. Phylogenetic analysis divided the proteins they encode into six clades (Figure S8). The two most divergent clades contained predominantly rice proteins. The OsRRA1 group is entirely comprised of rice proteins, while the OsRRA2 group, in addition to rice proteins, contains one representative from *A. trichopoda* and *N. colorata* each. The remaining four clades contained 2–5 tea RRA proteins each. Analysis of the aa sequences of RRAs (Figure S9) revealed that four CsRRAs (XP_028101801.1, XP_028078435.1, XP_028086375.1, and XP_028068574.1) from the ARR9, ARR3, and ARR16 clades are almost half the size (approx. 200 aa) compared to other RRAs. The remaining tea representatives retain conserved DD and KP sequences in the RD. Only XP_028054327.1 appears to be a PRR with asparagine instead of the phosphorylated aspartate.

Interestingly, the potential CsPRR, a member of the ARR16 group, has a TM domain at its C-terminus. Furthermore, a TM domain at the C-terminus is predicted for rice OsRRA9, and a representative of *N. colorata* (XP_031478630.2) has two putative TM domains at its N-terminus (Figure S9).

## 3. Discussion

In this work, we verified and extended the main results of our previous in silico analysis [32] by performing an experimental study of the TM-containing phosphotransfer protein from *C. sinensis*. For this, coding sequences of both isoforms of the CsHPt1 protein, TM-containing and soluble, were cloned from plant samples, and the localization of encoded proteins in plant cells was determined. Importantly, the mRNA templates for both CsHPt isoforms were proven to ensure the biosynthesis of full-sized phosphotransfer protein fusions in plant cells.

Confocal microscopy showed different patterns of distribution of putative soluble and TM-HPts in the cell. The subcellular localization of sol-CsHPt1 was in agreement with that of canonical soluble HPts [27,50]. Co-localization of the sol-CsHPt1 with the nuclear marker clearly demonstrated the nuclear localization. The partial overlap of the fluorescent signals of the sol-CsHPt1 with the PM marker observed in this study has previously been reported for canonical HPts [27]. This apparent localization may be caused by the large vacuole, which presses the cytosol and internal membranes against the PM. In the case of sol-CsHPt1, individual strands crossing the cell segments, which are visible only in the GFP fluorescence channel (Figure 2 and Figure S1), indicated that this protein is localized in the cytosol.

The subcellular localization pattern of TM-CsHPt1 was different compared to sol-CsHPt1. Importantly, the fluorescent signal of TM-CsHPt1 surrounded the nucleus and did not co-localize with the nuclear marker. The observed dense intracellular net in the GFP channel (Figure 2 and Figure S1), together with the perinuclear signal, indicated (at least partial) localization of this protein in the ER [27,51,52]. The apparent co-localization of TM-CsHPt1 with the PM marker may be artifactual for the reasons described above. To specify the subcellular localization of the studied proteins, the two-phase membrane partition followed by immunoblotting was run. This approach showed the occurrence of TM-CsHPt1 in the ER-enriched inner membrane fraction but not in the PM-enriched fraction (Figure 3).

Our results refute the obvious explanation regarding the role of TM-CsHPt1 in MSP, namely, that TM-CsHPt1 performs the functions of ordinary soluble CsHPt. For the latter, it would be sufficient to simply treat the TM domain with some exo- or endo-protease, which would eliminate the ability of TM to attach TM-CsHPt1 to the membrane. In this regard, the immunochemistry experiment (Figure 3) was of particular importance, showing that full-length TM-CsHPt1 was synthesized with no signs of degradation from the N-terminus. This is also evidenced by the virtually 100% presence of the TM-CsHPt1–GFP fusion in the membrane; otherwise, part of the TM-CsHPt1–GFP would be detected in the cytosol as well. These data are also important for explaining the results of the experiment studying the function of TM-CsHPt1 in plant cells (Figure 5). If TM were easily cleaved from TM-CsHPt1 in planta, this could well explain the lack of any effect of overexpression of the recombinant protein in tobacco leaves. So, at least this possible explanation does not hold in light of our results.

The fact that TM-CsHPt1 of *C. sinensis* was cloned only from the callus grown in the dark suggests the occurrence of this phosphotransfer protein in the underground plant organs, namely roots. Indeed, global transcriptomic studies of tea plants revealed the *TM-CsHPt1* transcripts just in roots [32]. The canonical *sol-CsHPt1* gene was expressed in the same callus line, but only in the illuminated cells. This suggests that light can influence the splicing of the corresponding gene, and two different splice forms are likely not present in the same cell simultaneously.

BiFC experiments confirmed the expected interaction of sol-CsHPt1 with all three Arabidopsis CK receptors (Figure 4). The subcellular pattern of their interaction with a clearly visible perinuclear signal is characteristic of the interaction of HPts with CK receptors [27]. Furthermore, the homodimerization of sol-CsHPt1 was detected. In this case, instead of circular fluorescence around the nucleus, fluorescence of the “filled” nucleus is observed, which is typical for canonical HPts dimers [27,29]. The features of sol-CsHPt1 protein–protein interactions (promiscuity, dimerization ability, and fluorescence patterns) characterize it as a classical HPt protein. These data confirm the possibility of obtaining relevant results in the system with tea HPts and Arabidopsis CK receptors.

**Figure 9:**
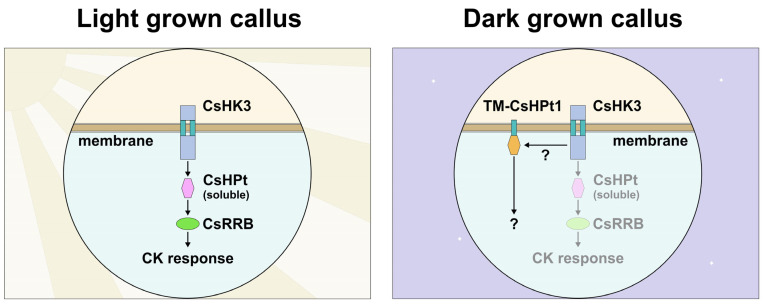
Possible function of the TM-containing phosphotransfer protein in tea cells. The scheme on the left depicts cytokinin signal transduction in callus cells cultured under daily lighting. The scheme on the right shows a putative membrane branch from the main CK signaling pathway due to the presence of a TM-CsHPt on the membrane (probably ER). The unknown interaction mechanism and non-canonical target of the membrane branch are indicated by question symbols.

Unlike sol-CsHPt1, TM-CsHPt1 has been shown to interact exclusively with the AHK3 receptor. It is important to note that CK receptors in different plant species are mainly localized in the ER [38,41,53,54,55]. Accordingly, the proximity of TM-CsHPt1 to the CK receptors on the ER membrane can be expected. However, the detectable specificity of interaction cannot be explained by the TM-CsHPt1 structure, since sol-CsHPt1 with the same interaction interface was able to interact with all three CK receptors. There is probably a certain feature of the AHK3 receptor that, unlike other CK receptors, allows it to interact with TM-HPt. Notably, in existing models of the interaction of the CK receptor with canonical HPt, RD is associated with other cytosolic domains of the receptor [43]. However, it is possible that the conformational mobility of the AHK3 RD is higher than in other receptors, favoring the AHK3 interaction with TM-HPt. Such “flexible” RDs of HKs are known, for example, in prokaryotes [56]. Of course, other reasons for the AHK3 specificity (differences in surface charge, hydrophobicity, or key aa residues in the receiver domains of AHK3 versus AHK2/AHK4) cannot be excluded either. In any case, the details of TM-CsHPt1 interaction with CK receptors and its functionality require further study, especially with domestic receptors (Figure 9).

Similar to canonical soluble HPts, TM-CsHPt1 can form homodimers. Surprisingly, we were unable to detect heterodimerization between TM-CsHPt1 and sol-CsHPt1, despite the structural identity of their dimerization interfaces. It can be assumed that the presence of the TM domain prevents their interaction, although the molecular mechanism for such prevention remains unclear.

Thus, our experimental results indicate that TM-CsHPt1 is presumably expressed in roots, localizes preferentially on the ER membrane, and can interact with AHK3 but not with any other two Arabidopsis CK receptors. It should be noted that the AHK3 receptor differs significantly in its ligand specificity from the AHK2 and AHK4 receptors, the latter being similar to each other [41,57,58,59,60]. The AHK3 receptor and its orthologs were shown to be expressed in all plant organs, including roots, and have a relatively high affinity to dihydrozeatin. These receptors were reported to implement specific functions in the root, namely, they determine root meristem size by controlling cell differentiation through affecting PIN-dependent auxin transport [61,62]. AHK3, alone or together with AHK2, plays a dominant role in cross-talk with other phytohormones (auxin, ethylene, and brassinosteroids) during the formation of lateral roots in Arabidopsis [63].

The selective interaction between TM-CsHPt1 and AHK3 orthologs may underlie the specificity of possible signal transduction involving these participants. Moreover, dimerization of HPts is also suggested to be important for specific signal transduction [14]. The failure of heterodimerization between TM-CsHPt1 and soluble CsHPt1 may also ensure the specificity of membrane signaling [32].

On the other hand, using artificial model systems (transiently transformed tobacco) may be impractical for studying the peculiar function of tea TM-HPt1, as the putative non-canonical signaling pathway may only exist in plants of the same species. Therefore, studying TM-CsHPt1 from *C. sinensis*, as well as other potential TM-HPt proteins in their natural hosts, is important for understanding the functions of this protein type.

To advance our understanding of the functioning of the CK signaling in *C. sinensis* plants, we characterized all key components of the tea CK system bioinformatically. Our results significantly expand and complement previously published data [64].

Gene multiplication is observed for virtually all participants in the tea CK signaling pathway. Each ortholog of the Arabidopsis receptors is encoded in the tea plant by two genes (Figure 6, Table S2). Multiplication is also observed for all *CsHPts* with the exception of the member of the AHP6 clade (Figure 7). A similar situation is typical for tea RRs of both types (Figures S6 and S8). Moreover, seven *CsRRBs* encode proteins that form a separate RR-clade (Figure S6). In such pairs or groups, genes often exhibit significant variability in exon-intron structure (Tables S2 and S3). These changes can result in both degradation and neofunctionalization of genes. In particular, among *CsHPts* in the AHP2,3,5 clade (Figure 6 and Figure 7), only one of the four coding sequences encodes a potentially functional protein.

Previously, following a phylogenetic analysis of potentially active TM-HPts in over 60 plant species, we concluded that TM-HPts have arisen independently multiple times. This is also supported by the significant diversity in the sequences and positions of predicted TM domains in phosphotransfer proteins of different plant species [32]. It looks like that plant species with a trend toward gene multiplication also has a tendency toward the emergence of TM-HPts. It should be noted that the emergence of TM-HPt is not necessarily the first event in the formation of a TM branch of signal transduction. On the contrary, TM-HPt can be integrated into some existing branch. For example, in rice, a new mechanism for CK signaling attenuation was recently described based on the recruitment of soluble OsHPts to the cell membrane [65,66].

The next objective of this study will be to determine whether the newly discovered TM-HPts function in signal transduction. In particular, whether their interaction with the receptor results in the transfer of the phosphoryl group.

The question of what provides specificity to MSP signaling has remained open for many years [14,16,67]. Apparently, compartmentalization of individual signaling components, as well as details of their metabolism, is one of the ways to ensure this specificity. Newly discovered TM-HPts require further research, especially in their host plants. Furthermore, the search for and study of other membrane components of MSP and, particularly, CK signaling are relevant. All this may provide new insight into the organization of the MSP system, helping to create plants with desired beneficial properties.

## 4. Materials and Methods

### 4.1. Plant Material and Growth Conditions

Leaves, leaf buds, and callus tissues of *C. sinensis* were used in this study. Samples of leaves and leaf buds (∼100 mg) were collected from intact *C. sinensis* plants growing in a greenhouse (Stock Greenhouse of the N.V. Tsitsin Main Botanical Garden of the Russian Academy of Sciences, Moscow) and immediately frozen in liquid nitrogen.

Callus tissues, grown in vitro, were previously obtained from the stem of young shoots of *C. sinensis* ‘Georgian’. Calli were grown on Heller’s nutrient medium containing 2,4-dichlorophenoxyacetic acid (5 mg/L), glucose (25 g/L), and agar (7 g/L) [68] and grown in the climatic chamber at +26 °C, with relative air humidity of 70%. Calli were grown for 40 days either in the dark or in a 16/8 h light/dark regimen with light intensities of 75 µmol·m^−2^·s^−1^.

### 4.2. RNA Isolation and cDNA Synthesis

Total RNA was extracted from frozen tissues using the ExtractRNA reagent (Evrogen, Moscow, Russia). The resulting RNA pellet was dissolved in 30 μL of RNase-free distilled water and treated with DNase I (Thermo Scientific, Waltham, MA, USA) to remove genomic DNA contaminants. Synthesis of cDNA was performed with the MMLV Reverse Transcriptase Kit (Evrogen, Moscow, Russia) according to the manufacturer’s instructions. The cloned samples were confirmed by sequencing and were stored at −24 °C.

### 4.3. Constructs for Plant Transformations

Several expression vectors have been constructed for the transient transformation of plants. The region of *pB7FWG2* containing *GFP* and the terminator was amplified using primers BcuIEcoRI Linker F and pB7 Modific R (Table S1). Then, *pB7FWG2* and the amplified product were treated with restriction enzymes BcuI and KpnI, and the amplified product was ligated into the specified vector using restriction sites. As a result, a plasmid with a linker for inserting the target sequence into the BcuI and EcoRI sites and combining it with GFP at the C-terminus of the target coding protein (*pB7–GFP*) was obtained.

cDNA sequences of TM-CsHPt1 (XP_028076088.1) and sol-CsHPt1 (XP_028076087.1) were amplified with Phusion High-Fidelity DNA Polymerase (Thermo Scientific, USA) and cloned into pJET1.2/blunt using the CloneJET PCR Cloning Kit (Thermo Scientific, USA). Primers for their cloning (TM-CsHPt1 F, sol-CsHPt1 F, and HPt R) are given in Table S1. Then, *HPt* sequences were subcloned into the *pB7–GFP* vector by the BcuI and EcoRI restriction sites using TM-CsHPt1 BcuI F, sol-CsHPt1 BcuI F, and HPt EcoRI R primers. In the constructs obtained, each *HPt* gene was fused to the constitutive *35S CaMV* promoter at the 5′-end and to the *GFP* at the 3′-end.

It is important to note that both potential splice forms of the tea CsHPt (LOC114278265) have different first exons. Therefore, the short form is not part of the longer one, and the cloned sequences are individual RNAs. The very fact that they were amplified from total cDNA proves the existence of alternative splicing during gene expression.

To create the plasmid with *mCherry*, the fluorescent protein sequence was amplified from *CD3-959* (*WAK2-mCherry-HDEL*) [69] using primers F1 mCherry and R mCherry. A fragment with a terminator was obtained from *pB7FWG2* using primers F mCherry-C and pB7 modific R. Both fragments were combined, and the resulting product was amplified using primers BcuIEcoRI Linker F and pB7 modific R. It was then inserted into pB7FWG2 at the BcuI and KpnI restriction sites. The resulting plasmid *pB7–mCherry* contained a linker for inserting the target sequence and combining it with *mCherry* at the C-terminus.

A construct with a plasma membrane marker was created. Using cDNA obtained from Arabidopsis leaves, we cloned the extracellular sequence of AHK1 flanked by TM domains using primers TM-AHK1 F and TM-AHK1 R. Then, it was cloned into *pJET1.2/blunt* using the CloneJET PCR Cloning Kit and then subcloned into *pB7–mCherry* using PCR with primers TM-AHK1 BcuI F and TM-AHK1 EcoR1 R. To create a nuclear marker, we used cDNA obtained from Arabidopsis leaves. From this cDNA, we cloned the *IAA17 (AXR3)* sequence using primers IAA17 F and R. Using PCR with primers F AXR3 P88L and R AXR3 P88L, a mutation was introduced into *IAA17* that makes this protein non-degradable in the proteasome. The sequence *IAA17 P88L* was subcloned into *pB7–mCherry* using PCR with primers IAA17 F BcuI and IAA17 R EcoR1.

To create the *pARR5–mCherry* construct, the *35S* promoter was removed from the *pB7–mCHERRY* plasmid using the restriction enzymes SacI and BcuI. The *ARR5* gene promoter sequence, amplified from the total genomic DNA of Arabidopsis, was inserted in its place using the primers pARR5 SacI F and pARR5 BcuI R. The correctness of all constructs was confirmed by DNA sequencing.

For BiFC experiments, *HPt* coding sequences were subcloned into *35S-pSPYNE* and *35S-pSPYCE* vectors [70] using TM-CsHPt BcuI F, sol-CsHPt BcuI F, and HPt Cfr9I R primers. In the constructs obtained, each target sequence was fused to the constitutive *35S CaMV* promoter at the 5′-end and to the N- or C-fragments of *eYFP* at the 3′-end. In these experiments, interactions were examined with the expression of each target sequence for both split eYFP variants. Controls with empty vectors lacking the target sequence were also included *(pSPYNE-35S–empty* + *pSPYCE-35S–empty*; *pSPYNE-35S–empty* + *pSPYCE-35S–AHK2*; *pSPYNE-35S–empty* + *pSPYCE-35S–sol-CsHPT1; pSPYNE-35S–empty* + *pSPYCE-35S–TM-CsHPT1*). The cloned samples were confirmed by sequencing.

### 4.4. Transient Expression of Constructs in Planta

All constructs described above were used for *Agrobacterium tumefaciens* transformation (strain GV3101) and then transiently expressed in *Nicotiana benthamiana* leaves. Transient transformation of *N. benthamiana* tobacco leaves was carried out according to [40]; 8-week-old plants grown under optimal temperature, watering, and light/darkness conditions were used. Infiltration was made with a mixture of *A. tumefaciens* clones harboring recombinant plasmids: the clone carrying the target construct(s) (OD600~0.035) and the clone carrying the *p19* gene (OD600~0.05) [71].

The expression of *p35S–TM-CsHPt1–eGFP*, *p35S–sol-CsHPt1–eGFP*, *p35S–IAA17_P88L–mCherry*, *p35S–TM-AHK1–mCherry*, and *p35S–TM-AHK1–eGFP* was detected 4–5 days after infiltration using a Nikon Ti2 Eclipse AX confocal microscope. All confocal laser scanning microscopy images were obtained using a Plan APO λD40X/0.95 objective without water immersion at constant imaging conditions. The GFP signal was acquired by excitation at 488 nm and detection in the 503–541 nm range. The mCherry signal was acquired by excitation at 561 nm and detection in the 599–630 nm range. The resulting confocal images were processed with NIS-Elements Viewer software (ver. 5.21.00).

In addition, a transient expression of all constructs was detected using a fluorescence microscope AxioImager Z2 (Carl Zeiss Microscopy GmbH, Göttingen, Germany) 4–5 days after infiltration. If required, leaves with transiently expressed (checked by fluorescence) constructs were then fractionated and used for protein immunoblot.

### 4.5. Membrane Fraction Isolation

All procedures of protein extraction were carried out at 4 °C. Total membrane fraction isolation was performed according to the method described in [58] with modifications [72]. Tobacco leaves expressing target sequences after removing the central vein were ground with a mortar and pestle in homogenization buffer (100 mM Tris-HCl, pH 7.6, 150 mM NaCl, 5 mM EDTA, 10 mM dithiothreitol (DTT), 1 mM phenylmethylsulfonyl fluoride (PMSF), and 0.5% sodium dodecyl sulfate). The buffer-to-plant material ratio was 3:1 *v*/*w*. The obtained homogenate was filtered through Miracloth (Calbiochem^®^, Gibbstown, NJ, USA). The filtrate was centrifuged at 10,000× *g* for 5 min, and the resulting supernatant was centrifuged at 18,000× *g* for 30 min. The membrane pellet was carefully resuspended in a 50 mM KCl + 10% glycerol solution. The fractions were stored at −70 °C.

PM- and ER-enriched membrane fraction isolation was performed as described in [41] with minor modifications. Briefly, transiently transformed tobacco leaves were homogenized, filtered through Miracloth (Calbiochem^®^), and stepwise centrifuged at 10,000× *g* and then at 100,000× *g.* The membrane pellet was carefully resuspended in phase buffer (5 mM K_2_HPO_4_/KH_2_PO_4_, pH 7.8, 0.33 M sucrose, 3 mM KCl, 0.1 mM EDTA) until individual vesicles. Membrane fractionation was implemented in an aqueous polymer two-phase system based on dextran 500,000 and PEG 3350. Separation was carried out in 36 g of a solution with an equal polymer concentration of 7.2% *w*/*w*.

The resulting composition was mixed, and the phases were separated by centrifugation at 3000× *g* for 10 min in a bucket rotor. The upper and lower phases were purified twice using similar pure polymer solutions. Purified compositions were mixed, and the phases were separated by centrifugation at 3000× *g* for 10 min in a bucket rotor.

A total of 2 mL of the lower phase, containing all intracellular membranes, was diluted with 30 mL of phase buffer. The upper phase enriched with PM was mixed with 1.224 g of KH_2_PO_4_ and 0.784 g of K_2_HPO_4_. After the salts were dissolved, the mixture was centrifuged at 3000× *g* for 10 min in a bucket rotor. Herewith, membranes moved to the interphase, and it was then diluted with 30 mL of the phase buffer. The resulting mixtures were centrifuged at 100,000× *g* for 30 min. The obtained precipitates were resuspended in 0.5 mL of 50 mM KCl + 10% glycerol solution.

### 4.6. Protein Immunoblot

Protein extracts (2 μg of total protein of each probe) were mixed with gel loading buffer. The proteins were separated by SDS electrophoresis in a 10% polyacrylamide gel and transferred onto a polyvinylidene fluoride membrane. Thereafter, blotting was made with primary rabbit polyclonal antibodies against GFP (Evrogen, Moscow, Russia) at a 1:2000 dilution. Membrane blocking was made for 1 h in phosphate-buffered saline (PBS) with 0.05% of Tween with 5% (PBST) with skim milk powder. The primary antibodies were incubated for 2 h in PBST. Then the membrane was washed three times in PBST. Tightly bound primary antibodies were visualized by goat secondary antibodies against rabbit immunoglobulins, conjugated to horseradish peroxidase (Millipore, Darmstadt Germany, AP132P). Incubation with secondary antibodies was performed at a 1:30,000 dilution in PBS for 1 h, followed by washing three times in PBST. Peroxidase activity was revealed with thte Ultra-Sensitive ECL Chemiluminescence Kit (Servicebio, Wuhan, China), and the product was detected using a blot scanner (LI-COR, Lincoln, NB, USA).

### 4.7. Bioinformatic Studies

The search for nucleotide/polypeptide sequences of phosphotransmitters was performed in the NCBI databases (http://www.ncbi.nlm.nih.gov, accessed on 11 March 2026) by using the BLASTP tool and the AHP1 (At3g21510) protein as a query sequence. They were aligned using Clustal Omega (https://www.ebi.ac.uk, accessed on 11 March 2026, clustalo 1.2.4). Phylogenetic trees were constructed using MrBayes-3.2.7. Clustal alignments were used as input, and Bayesian MCMC phylogenetic trees were constructed based on Markov chain Monte Carlo simulation with the general time reversible (GTR) nucleotide substitution model and site-rate variation drawn from a discrete gamma distribution with six classes. A total of 1,000,000 generations were taken to reach a standard deviation of split frequencies below 0.01. Statistical support of phylogenetic trees was estimated primarily through Bayesian posterior probabilities of correctness of particular nodes. The resulting tree was visualized by FigTree v1.4.4 (http://tree.bio.ed.ac.uk/software/figtree, accessed on 11 March 2026). The domain structure of proteins was determined using the InterPro web service (https://www.ebi.ac.uk/interpro/, accessed on 11 March 2026). Transmembrane domains were determined using TMHMM 2.0 (https://services.healthtech.dtu.dk/services/TMHMM-2.0/, accessed on 11 March 2026) and Phobius (https://phobius.sbc.su.se/, accessed on 11 March 2026) algorithms for the presence of predicted TM domains.

Visualization of protein sequences was carried out by Clustal X2.1 [73] and Jalview [74].

Molecular modeling by the de novo method was performed using the IntFOLD (version 7.0) web service (https://www.reading.ac.uk/bioinf/IntFOLD/, accessed on 11 March 2026) [75]. The models were optimized and embedded in an artificial membrane using the YASARA Structure software (version 22.9.24) [76]. Models were additionally optimized and visualized in UCSF Chimera software (version 1.14) [77].

## 5. Conclusions

Here, we present the first experimental study of non-canonical transmembrane phosphotransfer phytoproteins. The expression of the gene encoding TM-HPt1 in intact *C. sinensis* cells was confirmed. This expression depends on the alternative splicing, which appears to be regulated by light. These data, together with the results of previously conducted transcriptomic analysis, indicate organ-specific (root) localization of the studied protein.

The uncovered transcripts were proven to be functional in encoding the full-sized TM-CsHPt1 protein with important consensus motifs, including those used for CK- and MSP signaling. The possible participation of TM-CsHPt1 in CK signaling is supported by its interaction with one of the three CK receptor paralogs, as well as its strict localization on the ER membranes, where most CK receptors reside. The lack of detectable interactions of TM-CsHPt1 with soluble CsHPt1 and some CK receptor paralogs raises new issues that need to be addressed using both in vivo and in silico methods. Furthermore, the general question of the biological significance of HPts’ homodimerization remains unanswered, which also applies to TM-HPts.

Previously, the possibility of TM-HPt involvement in the negative regulation of CK signaling was suggested, but we have not found any experimental evidence to support this. On the other hand, we have established detailed molecular and phylogenetic characteristics of the cytokinin signaling system in tea *C. sinensis* cells. These findings will help to provide valuable insights into the putative roles of non-canonical components of the MSP system in plants.

## Data Availability

All data presented in this study are available in the article and Supplementary Materials.

## Supplementary Materials

The following supporting information can be downloaded at: https://www.mdpi.com/article/10.3390/ijms27104253/s1.

## Abbreviations

The following abbreviations are used in this manuscript:

aa	Amino acid
CK	Cytokinin
DI	Dimerization interface
ER	Endoplasmic reticulum
HK	Histidine kinase
HPt	Phosphotransmitter
MSP	Multistep phosphorelay
PHP	Pseudo-HPt
PRR	Pseudo response regulator
RD	Receiver domain
PM	Plasma membrane
REC-like	receiver-like (domain)
RR	Response regulator
RRA	A-Type response regulator
RRB	B-Type response regulator
SM	Sensor module
sol-HPt	Soluble phosphotransmitter
TM	Transmembrane domain (or protein)
TM-HPt	TM-containing phosphotransmitter
