# Supplementary material for: An Unusual Component of the Multistep Phosphorelay from Tea Plant (Camellia sinensis L.)
Source: Int J Mol Sci. 2026 May 10;27(10):4253. doi: 10.3390/ijms27104253 (PMC13206810; doi:10.3390/ijms27104253)
Supplement: Supplementary file 1 [file ijms-27-04253-s001.zip › ijms-4266916-supplementary.pdf]

Supplementary data

Savelieva et al.

Table S1. Primers used in the study

**Primer name**	**Primer sequences (5 – 3)**
TM-CsHPt1 F	5`-ATG GGT TTT GTT TTG TTT TTG TTG TTT TGG-3`
sol-CsHPt1 F	5`-ATG GAG GTG GGT GAG TTG CA-3`
CsHPt1 R	5`-TTC CAT CAG GGG AAT TGA CCC-3`
TM-CsHPt1 BcuI F	5`-TAT ACT AGT ATG GGT TTT GTT TTG TTT TTG TTG TTT TGG-3`
sol-CsHPt1 BcuI F	5`-TAT ACT AGT ATG GAG GTG GGT GAG TTG CA-3`
CsHPt1 EcoRI R	5`-TAT GAA TTC TTC CAT CAG GGG AAT TGA CCC-3`
CsHPt1 Cfr9I R	5`-TAT CCC GGG TTC CAT CAG GGG AAT TGA CCC-3`
BcuIEcoRI Linker F	5`-TGA CTA GTC TTT CTT GAA TTC AAC CCA GCT TTC TTG TAC AA-3`
pB7 modific R	5`-ACG TGA CTC CCT TAA TTC TC-3`
F1 mCherry	5`-ACC CAG CTT TCT TGT ACA AAG TGG TGA TAT CAA TGG TGA GCA AGG GC-3`
R mCherry	5`-TTA CTT GTA CAG CTC GTC CA-3`
F mCherry-C	5`-TGG ACG AGC TGT ACA AGT AA-3`
IAA17 F BcuI	5`-ATA ACT AGT ATG GGC AGT GTC GAG C-3`
IAA17 R EcoR1	5`-TCT TGA ATT CAG CTC TGC TCT TGC AC-3`
TM-AHK1 BcuI F	5`-ATC ACT AGT ATG GTC TTT GTT GTT CGC CTC-3`
TM-AHK1 EcoR1 R	5`-CTT GAA TTC TGT GAG TAT TAA AAT GCA G-3`
TM-AHK1 F	5`-ATG CGA GGA GAT AGC TTC TC-3`
TM-AHK1 R	5`-TGT GAG TAT TAA AAT GCA GAC AC-3`
F AXR3 P88L	5`-GGA TGG CCA CTG GTG AGA TCA TA-3`
R AXR3 P88L	5`-TATGATCTCACCAGTGGCCATCC-3`
pARR5 SacI F	5`-ATT ATT GAG CTC AAA ACA CCA TTT AGT CTA AAA TGT ATT T-3`
pARR5 BcuI R	5`- ATT ACT AGT ATC AAG AAG AGT AGG ATC GTG-3`
IAA17 F	5`-ATG GGC AGT GTC GAG C-3`
IAA17 R	5`-AGCTCTGCTCTTGCACTTCT-3`

Table S2. Key components of the cytokinin signaling pathway in *Camellia sinensis**

	Locus name	Protein ID	aa	mRNA ID	bp	Number of exons	Gene location
**Putative CK receptors**	LOC114256139	XP_028051540.1	988	XM_028195739.1	4154	12(11)	NW_021025386.1 (2297645..2309181)
		XP_028051544.1	834	XM_028195743.1	3377	10	
		XP_028051545.1	820	XM_028195744.1	3653	11(10)	
	LOC114280237	XP_028078389.1	979	XM_028222588.1	3876	11	NW_021026626.1 (2369732..2379915, complement)
	LOC114260337	XP_028056227.1	1250	XM_028200426.1	5141	14(13)	NW_021024599.1 (902601..913941, complement)
		XP_028056235.1	1249	XM_028200434.1	3750	13	
	LOC114258785	XP_028054579.1	1248	XM_028198778.1	5092	15(12)	NW_021025509.1 (396749..407811)
	LOC114293806	XP_028093747.1	1040	XM_028237946.1	4777	11(10)	NW_021024699.1 (1374437..1387606, complement)
		XP_028093742.1	1040	XM_028237941.1	4680	12(10)	
		XP_028093739.1	1040	XM_028237938.1	4823	11(10)	
	LOC114255909	XP_028051252.1	1040	XM_028195451.1	3965	10	NW_021025372.1 (2086461..2094145, complement)
		XP_028051253.1	880	XM_028195452.1	2792	10	
**Type B Response Regulators**	LOC114270834	XP_028068255.1	678	XM_028212454.1	3042	6	NW_021026086.1 (1298917..1304391, complement)
	LOC114275401	XP_028073237.1	657	XM_028217436.1	2273	6	NW_021026306.1 (613745..618981, complement)
	LOC114275402	XP_028073238.1	653	XM_028217437.1	2463	6	NW_021026306.1 (603401..617241, complement)
		XP_028073239.1	548	XM_028217438.1	2011	5(4)	
		XP_028073240.1	523	XM_028217439.1	2073	5	
	LOC114287248	XP_028086327.1	703	XM_028230526.1	2967	6	NW_021027134.1 (491880..498309)
	LOC114265222	XP_028061793.1	631	XM_028205992.1	4409	5	NW_021024614.1 (605061..610965)
		XP_028061797.1	594	XM_028205996.1	2271	5	
	LOC114268537	XP_028065519.1	572	XM_028209718.1	2205	5	NW_021025954.1 (52645..57402, complement)
	LOC114260366	XP_028056252.1	555	XM_028200451.1	1908	7	NW_021025588.1 (1465339..1517535, complement)
	LOC114282873	XP_028081437.1	472	XM_028225636.1	1934	7(6)	NW_021026831.1 (68439..73869, complement)
		XP_028081438.1	407	XM_028225637.1	1915	6(5)	
		XP_028081439.1	407	XM_028225638.1	1833	6(5)	
		XP_028081440.1	407	XM_028225639.1	1801	6(5)	
	LOC114310270	XP_028112021.1	455	XM_028256220.1	1701	6(5)	NW_021024888.1 (3217618..3222581, complement)
	LOC114288671	XP_028088032.1	577	XM_028232231.1	1734	8	NW_021024687.1

							(975031..989657)
	LOC114317244	XP_028119757.1	355	XM_028263956.1	1068	6	NW_021025118.1 (611118..628187, complement)
	LOC114258794	XP_028054587.1	377	XM_028198786.1	1134	4	NW_021025509.1 (67568..69198, complement)
	LOC114283644	XP_028082307.1	532	XM_028226506.1	2218	7	NW_021026886.1 (1754011..1759407)
	LOC114294842	XP_028094842.1	497	XM_028239041.1	1494	8	NW_021024508.1 (2353378..2358353)
	LOC114320755	XP_028123619.1	673	XM_028267818.1	2022	7	NW_021025237.1 (1983555..1989930, complement)
**Type A Response Regulators**	LOC114280284	XP_028052762.1	210	XM_028196961.1	1264	5	NW_021025424.1 (2297815..2300047, complement)
		XP_028052763.1	210	XM_028196962.1	1268	5	
	LOC114301092	XP_028101801.1	134	XM_028246000.1	514	1	NW_021029640.1 (91402..91915)
	LOC114306063	XP_028107020.1	210	XM_028251219.1	1137	4	NW_021024756.1 (70167..71826, complement)
	LOC114284056	XP_028082733.1	256	XM_028226932.1	1067	5	NW_021026912.1 (395746..398970)
	LOC114280284	XP_028078435.1	145	XM_028222634.1	1154	3	NW_021024659.1 (1253789..1255160, complement)
	LOC114270962	XP_028068391.1	297	XM_028212590.1	1463	5	NW_021026091.1 (153413..155418)
	LOC114258410	XP_028054170.1	226	XM_028198369.1	990	5	NW_021025491.1 (1412296..1414848)
	LOC114256227	XP_028051643.1	199	XM_028195842.1	970	4	NW_021025388.1 (1121148..1123741, complement)
	LOC114287278	XP_028086375.1	95	XM_028230574.1	762	5(4)	NW_021027137.1 (795840..799923)
		XP_028086376.1	95	XM_028230575.1	762	5(4)	
		XP_028086377.1	95	XM_028230576.1	753	5(4)	
	LOC114304097	XP_028105071.1	146	XM_028249270.1	1052	1	NW_021032881.1 (491..1042, complement)
	LOC114260670	XP_028056641.1	145	XM_028200840.1	625	4	NW_021025605.1 (697799..698698)
		XP_028056642.1	127	XM_028200841.1	570	4	
	LOC114258556	XP_028054327.1	200	XM_028198526.1	603	7	NW_021025497.1 (1488706..1493510, complement)
	LOC114317793	XP_028120351.1	205	XM_028264550.1	636	7	NW_021025135.1 (83053..88571)
	LOC114271159	XP_028068574.1	120	XM_028212773.1	363	4	NW_021024632.1 (624172..627484, complement)

* Phosphotransfer proteins of *C. sinensis* are presented in Table S3.

Number of exons in parentheses indicates the number of protein-coding exons. In the column « Gene location» reverse orientation of gene is indicated as complement. Genome assembly AHAU_CSS_1 was used to collect genomic data.

Table S3. Phosphotransfer proteins from *Camellia sinensis* and plant species used in phylogenetic and structural analysis.

Locus name	protein ID	aa	mRNA ID	bp	Exon num.	Gene location
Arabidopsis thaliana/TAIR10.1						
AHP1	NP_188788.1	154	NM_113046.4	1434	6	Chr3; NC_003074.8 (7577712..7579789, complement)
AHP2	NP_189581.1	156	NM_113860.4	878	6	Chr3; NC_003074.8 (11264126..11265562, complement)
AHP3	NP_001318703.1	155	NM_001344287.1	1177	6	Chr5; NC_003076.8 (15748731..15750891)
AHP5	NP_563684.1	157	NM_100225.3	1226	6	Chr1; NC_003070.9 (847793..849621)
AHP6	NP_001321526.1	160	NM_001334957.1	970	3	Chr1; NC_003070.9 (30133718..30134937)
AHP4	NP_001189912.1	145	NM_001202983.2	863	6	Chr3; NC_003074.8 (5554197..5555789)
Camellia sinensis/AHAU_CSS_1						
LOC114278265	XP_028076088.1	164	XM_028220287.1	849	5	NW_021026499.1 (1470368..1473201, complement)
	XP_028076089.1	151	XM_028220288.1	760	6	
LOC114278183	XP_028075990.1	151	XM_028220189.1	1008	6	NW_021026497.1 (225879..228571)
LOC114275608	XP_028073440.1	151	XM_028217639.1	739	6	NW_021024648.1 (456743..461775)
LOC114270089	XP_028067288.1	143	XM_028211487.1	752	6	NW_021026048.1 (631374..633517, complement)
LOC114323511	XP_028126924.1	159	XM_028271123.1	513	2	NW_021025341.1 (693080..693732, complement)
LOC114324070	XP_028127594.1	126	XM_028271793.1	414	2	NW_021025367.1 (2020788..2021314)
LOC114269493	XP_028066632.1	152	XM_028210831.1	1031	6	NW_021026004.1 (882502..885811, complement)
LOC114262616	XP_028058779.1	173	XM_028202978.1	932	6	NW_021025686.1 (445814..454989, complement)
	XP_028058780.1	156	XM_028202979.1	733	5	
	XP_028058781.1	153	XM_028202980.1	882	6	
	XP_028058782.1	149	XM_028202981.1	862	5	
LOC114270605	XP_028067929.1	157	XM_028212128.1	1279	5	NW_021026077.1 (328992..331986)
LOC114313369	XP_028115560.1	150	XM_028259759.1	808	6	NW_021024988.1 (120089..122875)
LOC114280519	XP_028078700.1	142	XM_028222899.1	796	6	NW_021026641.1 (944268..947888)
	XP_028078701.1	119	XM_028222900.1	729	5	
	XP_028078702.1	118	XM_028222901.1	726	5	
Solanum lycopersicum/SLM_r2.1						
LOC101244413	NP_001297079.1	151	NM_001310150.1	801	7(6)	Chr1; NC_090800.1 (75916327..75918544)

LOC101253938	XP_004230155.1	148	XM_004230107.5	1057	6	Chr1 ; NC_090800.1 (84797030..84798943, complement)
LOC101249559	XP_025887578.1	152	XM_026031793.2	4161	7(6)	Chr6; NC_090805.1 (51925729..51932808)
LOC101250875	XP_004235955.1	156	XM_004235907.5	907	5	Chr3; NC_090802.1 (62878142..62879817, complement)
LOC101249791	XP_004251188.2	137	XM_004251140.4	1130	6	Chr11; NC_090810.1 (60089883..60093157, complement)
LOC101248225	XP_010325073.1	136	XM_010326771.4	732	6	Chr8; NC_090807.1 (56327926..56331181, complement)
Lotus japonicus/LjGifu_v1.2						
LOC130723857	XP_057430982.1	154	XM_057574999.1	863	7(6)	Chr6; NC_080046.1 (62134297..62136186)
LOC130718227	XP_057424739.1	151	XM_057568756.1	797	6	Chr5; NC_080045.1 (52390673..52392191, complement)
LOC130732743	XP_057440717.1	154	XM_057584734.1	736	6	Chr1; NC_080041.1 (134850257..134851981)
LOC130720452	XP_057427079.1	152	XM_057571096.1	896	6	Chr5; NC_080045.1 (56889980..56894016)
LOC130749435	XP_057458771.1	146	XM_057602788.1	1398	4	Chr3; NC_080043.1 (979498..981444)
LOC130742588	XP_057450662.1	146	XM_057594679.1	1551	4	Chr3; NC_080043.1 (73773753..73775662)
LOC130748273	XP_057457439.1	150	XM_057601456.1	808	6	Chr3; NC_080043.1 (2237431..2239017)
LOC130742656	XP_057450742.1	151	XM_057594759.1	766	6	Chr3; NC_080043.1 (76121341..76123154)
Oryza sativa/AGIS1.0						
OsAHP1	NP_001390620.1	147	NM_001403691.1	1204	6	Chr8; NC_089042.1 (28051969..28056922)
OsAHP2	NP_001390867.1	149	NM_001403938.1	1159	6	Chr9; NC_089043.1 (27099074..27102271)
OsPHP1	NP_001388547.1	151	NM_001401618.1	3944	6	Chr1; NC_089035.1 (31689267..31694642)
OsPHP2	NP_001407161.1	151	NM_001420232.1	712	6	Chr5; NC_089039.1 (5298137..5300402, complement)
OsPHP3	NP_001407671.1	152	NM_001420742.1	1047	6	Chr5; NC_089039.1 (26045856..26053215, complement)
Amborella trichopoda/AMTR1.0						
LOC18425680	XP_006830280.2	151	XM_006830217.3	745	6	NW_006496517.1 (1603900..1605607, complement)
LOC18431914	XP_006842046.1	160	XM_006841983.2	702	5	NW_006498085.1 (88324..89365)
LOC18441356	XP_011625967.1	148	XM_011627665.2	966	6	NW_006499824.1 (2839511..2847699)
LOC18443432	XP_006853683.1	149	XM_006853621.3	729	6	NW_006499905.1 (1823949..1826145)

Number of exons in parentheses indicates the number of protein-coding exons. In the column « Gene location» reverse orientation of gene is indicated as complement.

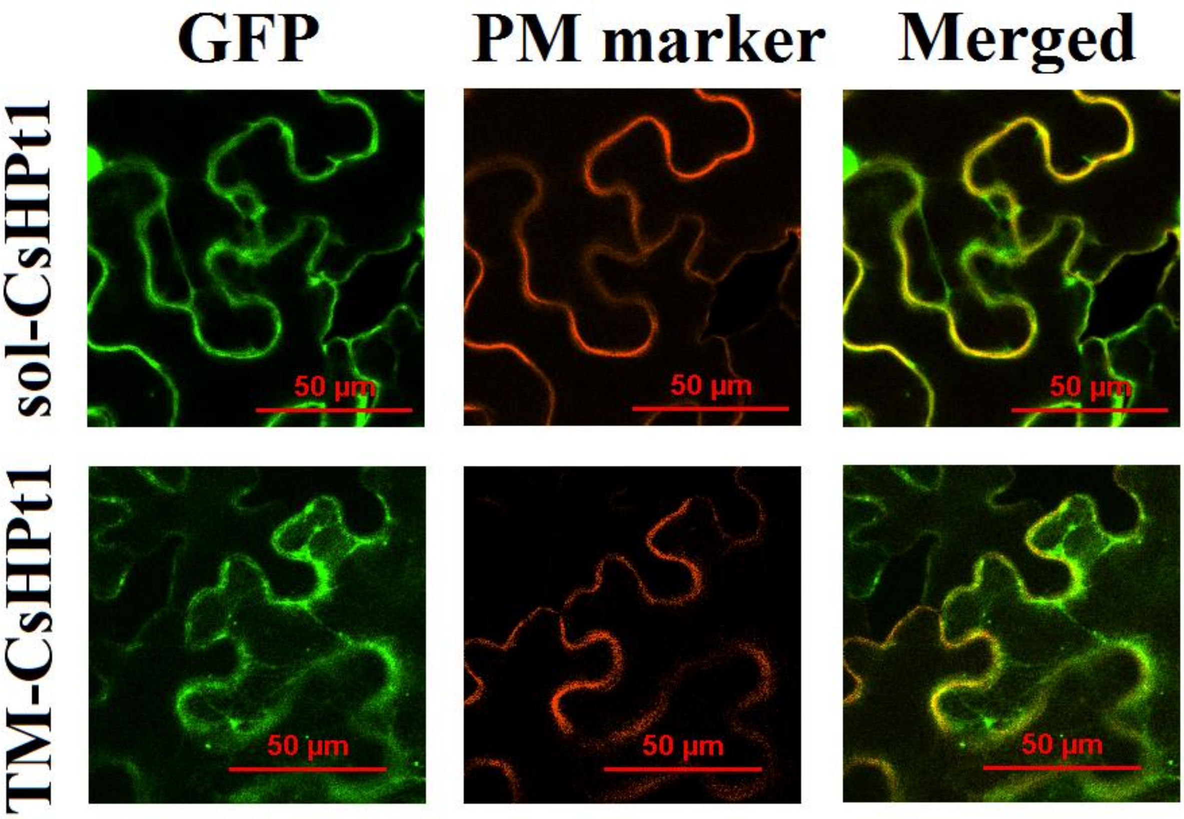

Figure S1. Co-localization of TM and soluble HPt isoforms of *C. sinensis* with the PM and nuclear marker proteins (high resolution), visualized by confocal microscopy. Bars = 50 µm. White arrows indicate nuclei. Inscriptions indicate: TM-CsHPt, TM-CsHPt−eGFP construct; sol-CsHPt1, sol-CsHP1t−eGFP construct; GFP, the column with eGFP fluorescence channel (shown in green); Marker, the column with mCherry fluorescence channel (shown in red); Merged, the column with merged channel. Yellow color indicates co-localization of the CsHPt proteins and the markers.

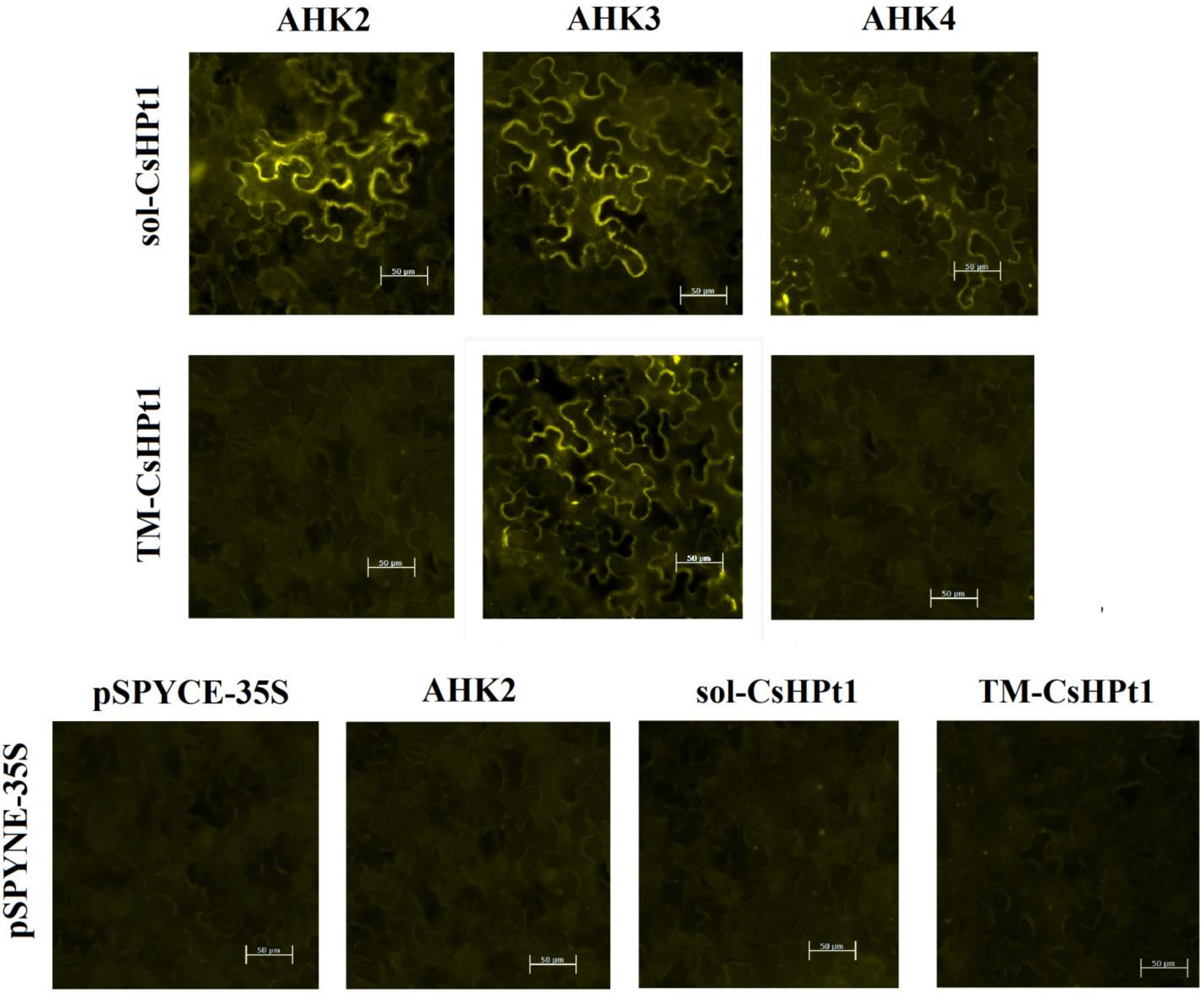

Figure S2. Interactions of CsHPt1 isoforms with CK receptors *in planta* determined by BiFC and visualized by fluorescence microscopy. Upper rows: vectors *pSPYNE-35S–AHK2–4*, *pSPYCE-35S–TM-CsHPt1*, *pSPYNE-35S–sol-CsHPt1* and *pSPYCE-35S–sol-CsHPt1*. Lower row: vectors *pSPYNE-35S–empty*, *pSPYCE-35S–empty*, *pSPYCE-35S–AHK2*, *pSPYCE-35S–sol-CsHPt1*, *pSPYCE-35S–TM-CsHPt1*. eYFP fluorescence is shown in yellow. Bars = 50 µm.

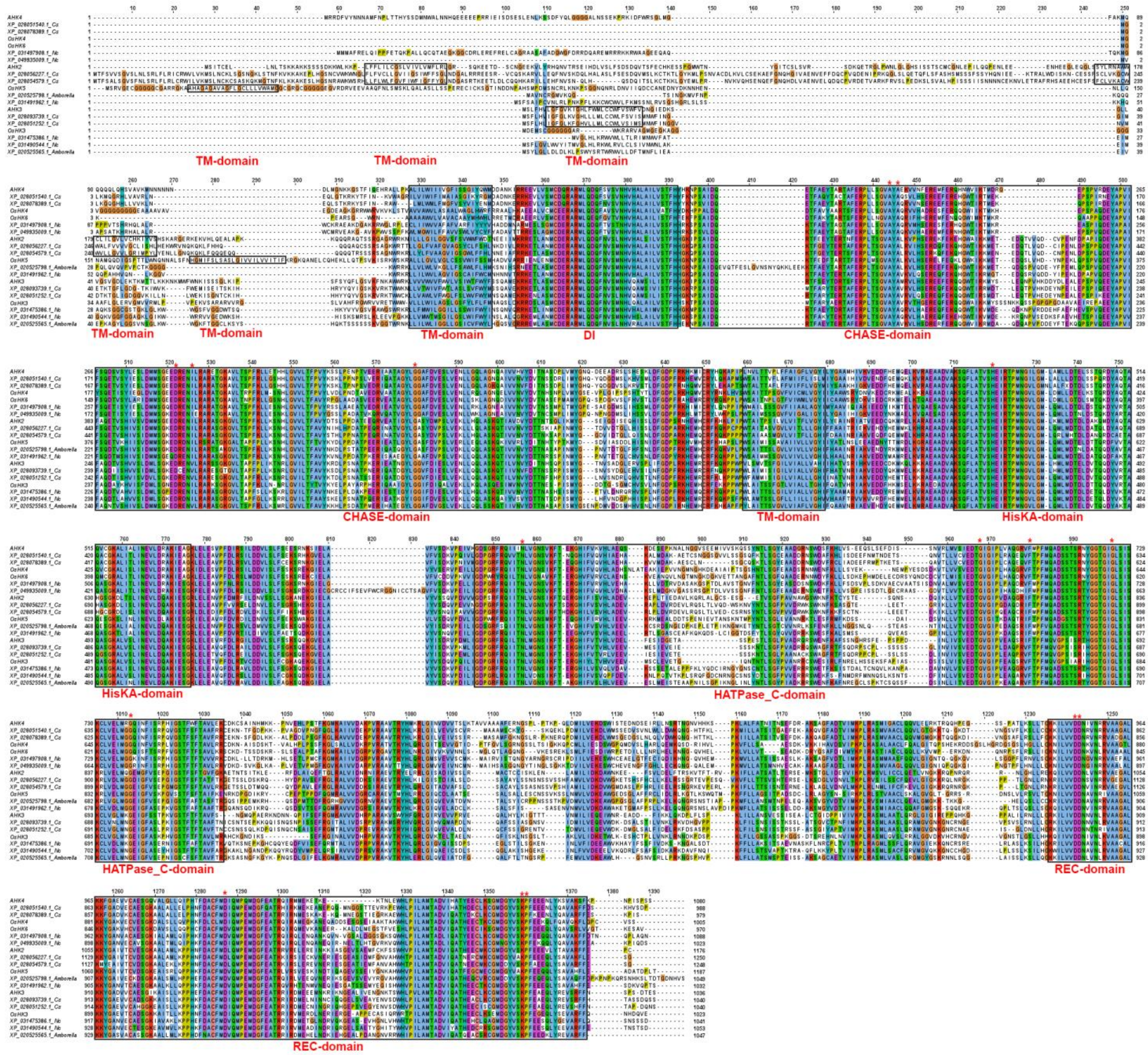

Figure S3. Alignment of the cytokinin receptor amino acid (aa) sequences (plant species as in Figure 6).

Black frames indicate crucial for function polypeptide structures. TM-domain, transmembrane domain; DI, dimerization interface region; CHASE-domain, Cyclase/Histidine kinase Associated SEnsory domain; HisKA-domain, histidine kinase dimerization domain, H-ATPase_C-domain, H-ATPase domain; REC-domain, receiver domain. Red asterisks indicate critical aa. The column on the left provide protein accession IDs and abbreviated plant species names or protein trivial names. Ambor, *Amborella trichopoda*; AHK2-4, Arabidopsis histidine kinase 2-4; Cs, *Camellia sinensis*; Nc, *Nymphaea colorata*; OsHK3-6, *Oriza sativa* histidine kinase 3-6

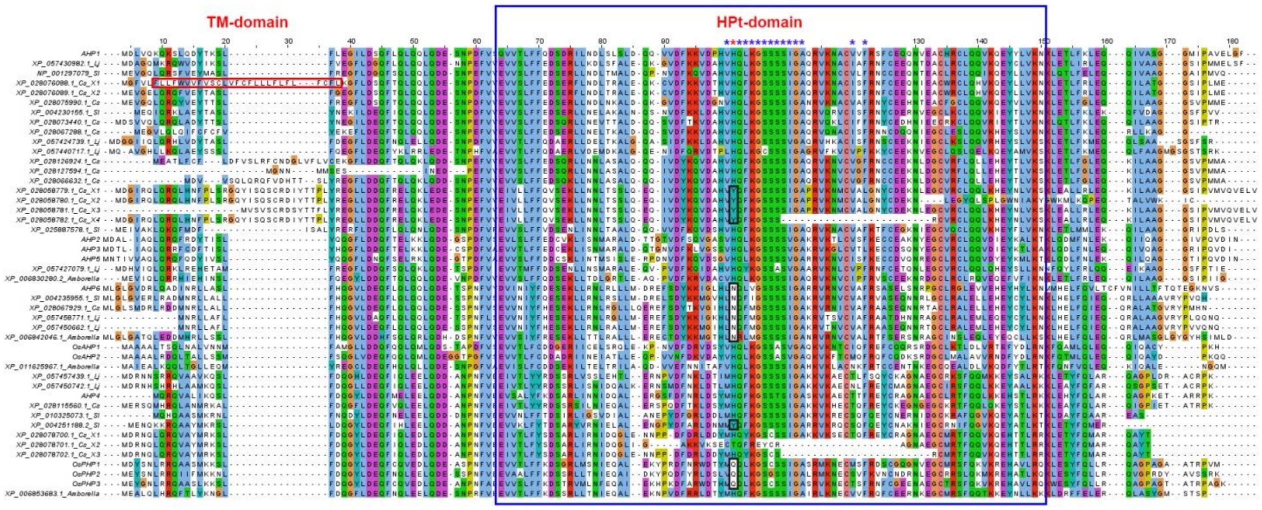

Figure S4. Alignment of the phosphotransfer protein aa sequences (plant species as in Figure 7).

TM-domain, transmembrane domain (marked with a red frame); HPt-domain, histidine phosphotransfer domain (marked with a red frame). Replacements of the conserved phosphorylatable histidine are indicated by black frames; the phosphorylatable motif is indicated by the blue asterisks, the phosphorylatable histidine is indicated by the red asterisk. The column on the left provide protein accession IDs and abbreviated plant species names or protein trivial names. *Amborella*, *Amborella trichopoda*; AHP1-6, Arabidopsis Histidine Phosphotransmitter1-6; Cs, *Camellia sinensis*; Lj, Lotus japonicus; *OsAHP1-2*, *Oriza sativa* Authentic Histidine Phosphotransmitter1-2; OsPHP1-3, *Oriza sativa* Pseudo Histidine Phosphotransmitter1-3; Sl, *Solanum lycopersicum*

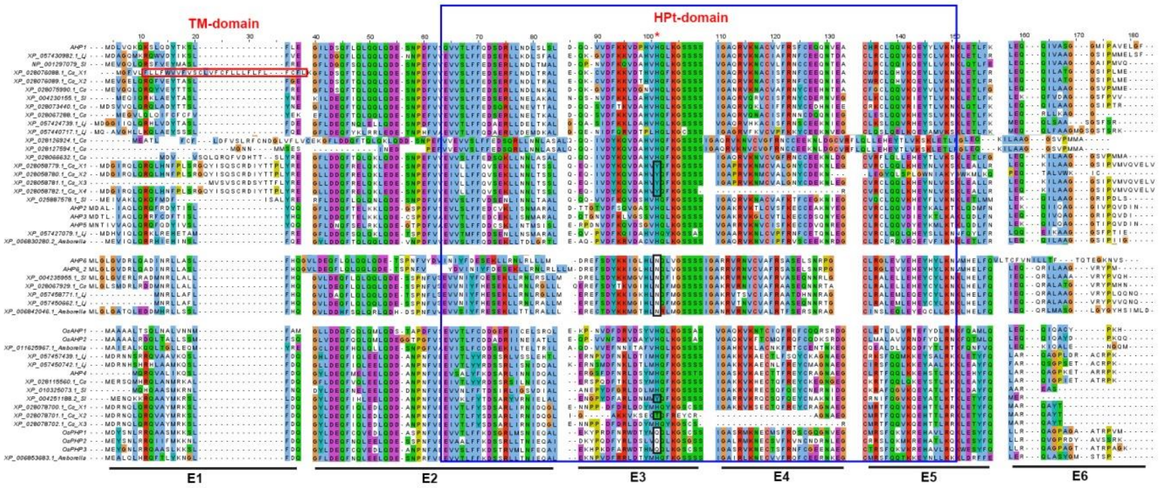

Figure S5. Alignment of the phosphotransfer protein aa sequences (plant species as in Figure 7) with division into exons.

TM-domain, transmembrane domain (marked with a red frame); HPt-domain, histidine phosphotransfer domain (marked with a red frame). Replacements of the conserved phosphorylatable histidine are indicated by black frames; E1-E6, exons of typical plant phosphotransfer protein gene structure. The column on the left provides protein accession IDs and abbreviated plant species names or protein trivial names. *Amborella*, *Amborella trichopoda*; AHP1-6, Arabidopsis Histidine Phosphotransmitter1-6; Cs, *Camellia sinensis*; Lj, Lotus japonicus; *OsAHP1-2*, *Oriza sativa* Authentic Histidine Phosphotransmitter1-2; OsPHP1-3, *Oriza sativa* Pseudo Histidine Phosphotransmitter1-3; Sl, *Solanum lycopersicum*

Two vertical gaps were introduced to form three groups with different exon-intron organizations while preserving the phylogenetic relationships of the sequences obtained from the phylogenetic analysis. In cases of deviation from the six-exon structure, the position of the third exon containing phosphorylatable histidine was retained unchanged, while another parts of sequences were moved left and right, maintaining the alignment structure.

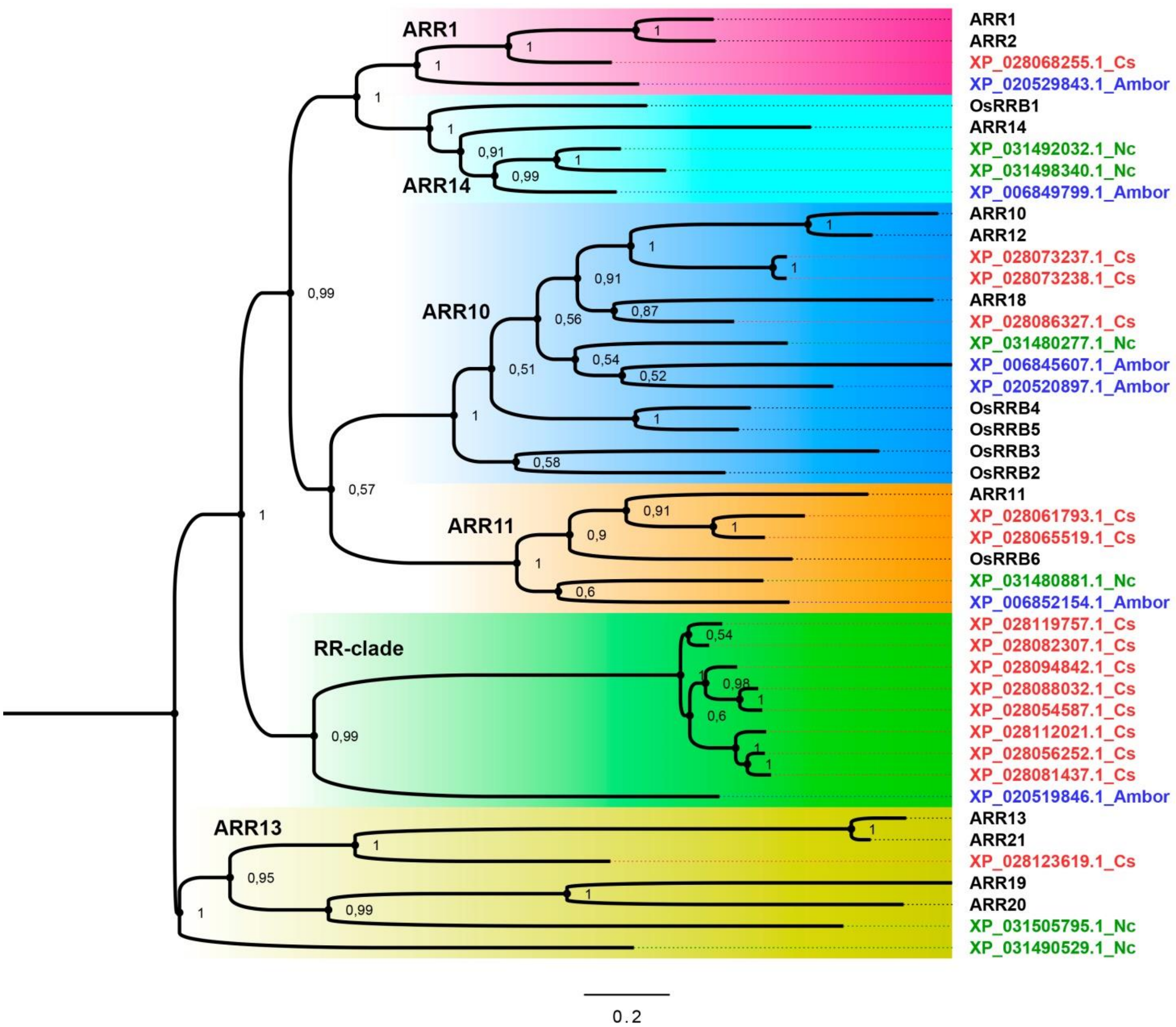

Figure S6. Phylogenetic tree of the B-type response regulators based on the protein sequences. This tree was constructed using MrBayes-3.2.7. The column on the right provide protein accession IDs and abbreviated plant species names or protein trivial names.

Ambor, *Amborella trichopoda*; ARR, Arabidopsis response regulator; Cs, *Camellia sinensis*; Nc, *Nymphaea colorata*; OsRRB, *Oriza sativa* B-type response regulator. The numbers at the base of the branches represent Bayesian posterior probabilities.

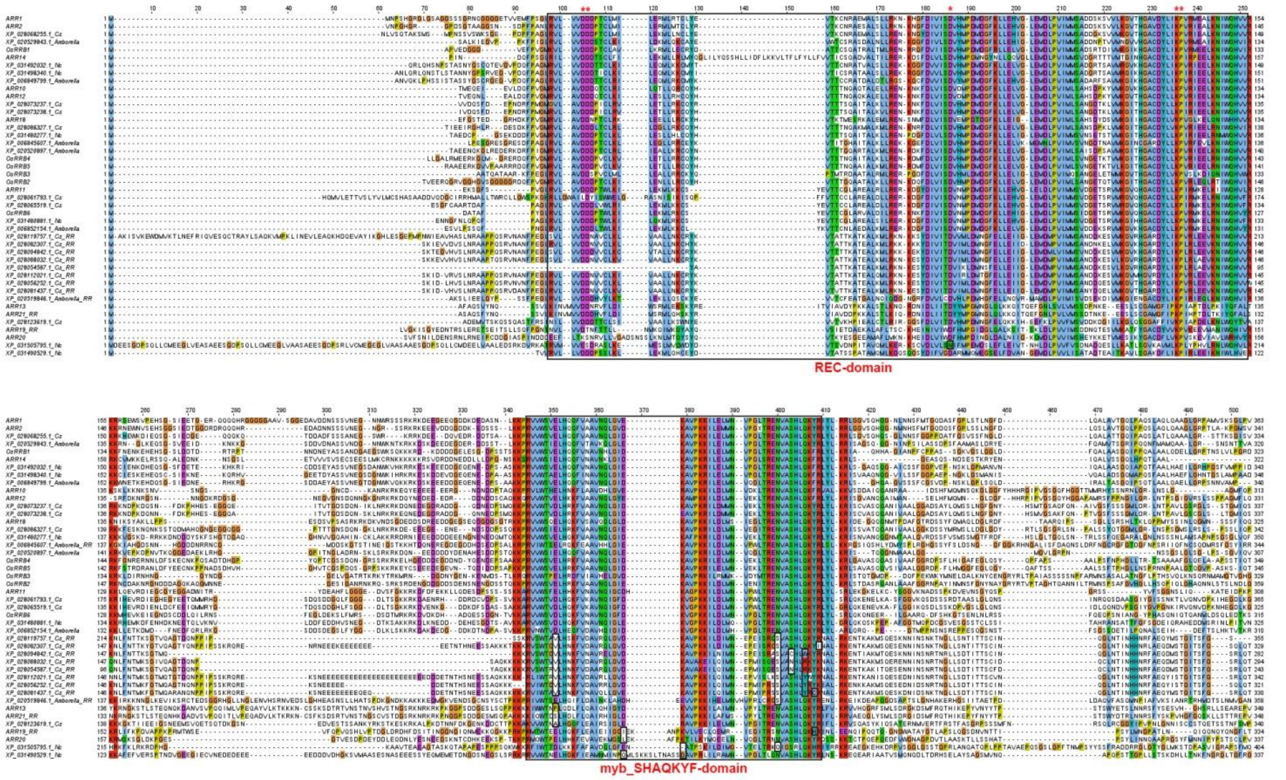

Figure S7. Alignment of the B-type response regulators amino acid sequences (plant species as in Figure S6) Amino acids highlighted in the black frames belong to the domain indicated below them.

REC-domain, receiver domain. Myb_SHAQKYF-domain, DNA-binding myb-like domain. The column on the left provide protein accession IDs and abbreviated plant species names or protein trivial names. Ambor, *Amborella trichopoda*; ARR, Arabidopsis response regulator; Cs, *Camellia sinensis*; Nc, *Nymphaea colorata*; OsRRB, *Oriza sativa* B-type response regulator. Red asterisks indicate aa critical for proper domain functioning.

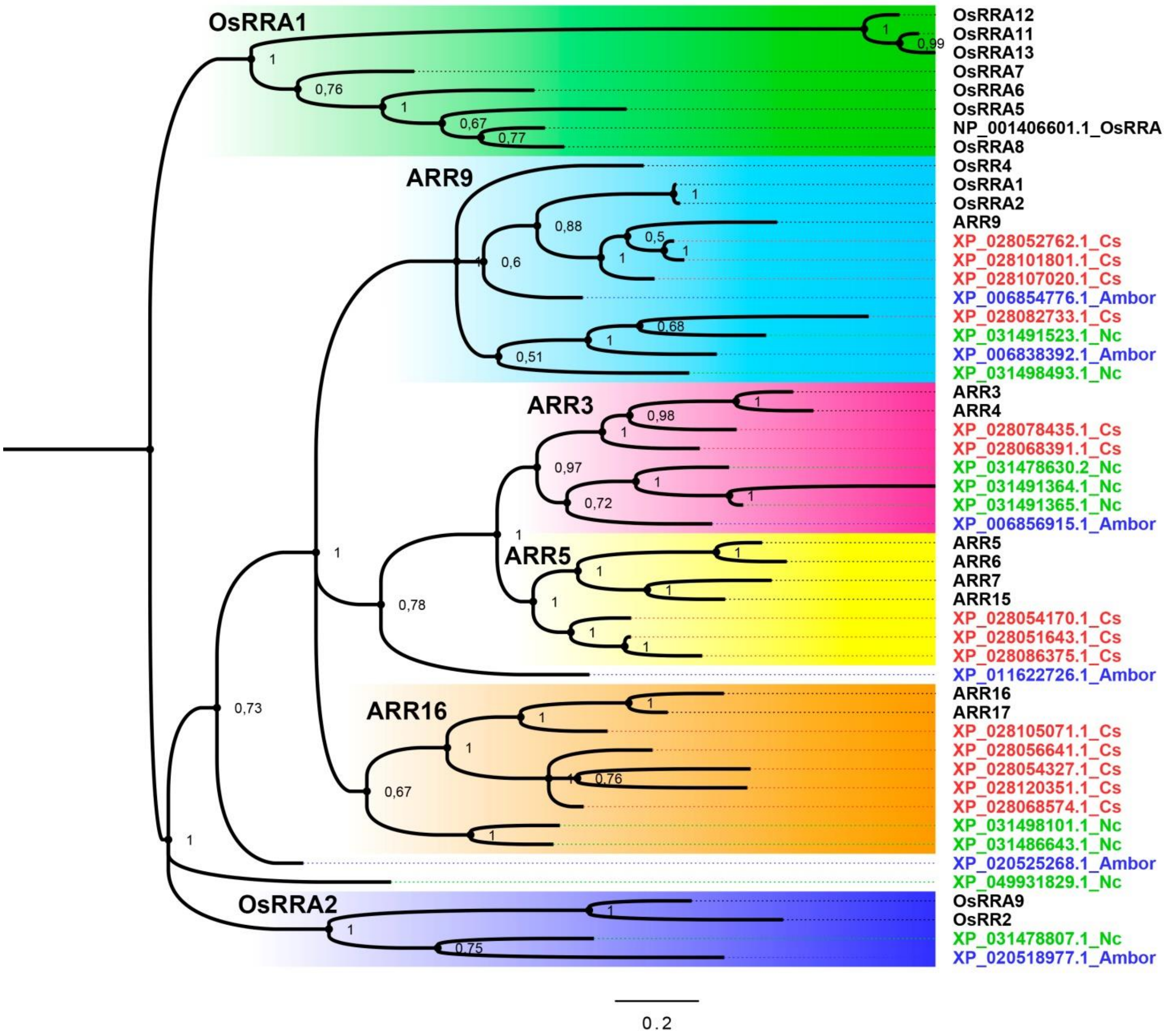

Figure S8. Phylogenetic tree of the A-type response regulators based on the protein sequences. This tree was constructed using MrBayes-3.2.7. The column on the right provide protein accession IDs and abbreviated plant species names or protein trivial names.

Ambor, *Amborella trichopoda*; ARR, Arabidopsis response regulator; Cs, *Camellia sinensis*; Nc, *Nymphaea colorata*; OsRR and OsRRA, *Oriza sativa* A-type response regulator. The numbers at the base of the branches represent Bayesian posterior probabilities.

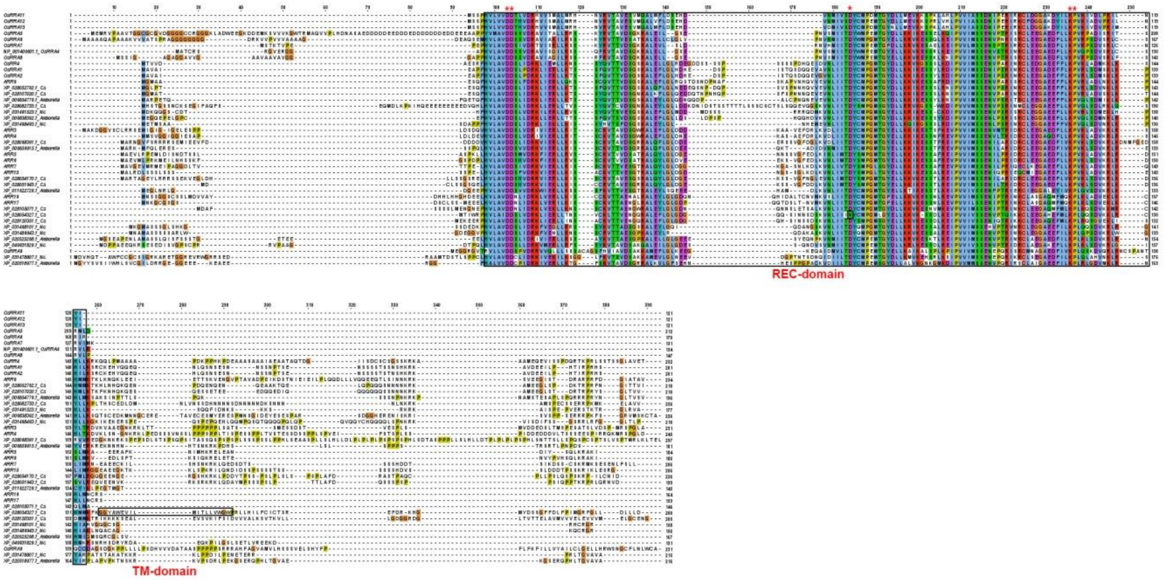

Figure S9. Alignment of the A-type response regulators amino acid sequences (plant species as in Figure S8)

Amino acids highlighted in the black frames belong to the domain indicated below them. REC-domain, receiver domain. Red asterisks indicate aa critical for proper domain functioning. The column on the left provide protein accession IDs and abbreviated plant species names or protein trivial names. Ambor, *Amborella trichopoda*; ARR, Arabidopsis response regulator; Cs, *Camellia sinensis*; Nc, *Nymphaea colorata*; OsRRB, *Oriza sativa* B-type response regulator.
